# Genetic Diversity and Signatures of Selection in 15 Chinese Indigenous Dog Breeds Revealed by Genome-Wide SNPs

**DOI:** 10.3389/fgene.2019.01174

**Published:** 2019-11-15

**Authors:** Qianyong Yang, Hao Chen, Junhua Ye, Chenlong Liu, Rongxing Wei, Congying Chen, Lusheng Huang

**Affiliations:** ^1^State Key Laboratory of Pig Genetic Improvement and Production Technology, Jiangxi Agricultural University, Nanchang, China; ^2^Jiangxi Provincial Key Laboratory for Police Dog Breeding and Behavioral Science, Nanchang Police Dog Base, Nanchang, China

**Keywords:** population structure, linkage disequilibrium, selection signatures, 170K SNP chip, Chinese indigenous dogs

## Abstract

There are dozens of recognized indigenous dog breeds in China. However, these breeds have not had extensive studies to describe their population structure, genomic linkage disequilibrium (LD) patterns, and selection signatures. Here, we systematically surveyed the genomes of 157 unrelated dogs that were from 15 diverse Chinese dog breeds. Canine 170K SNP chips were used to compare the genomic structures of Chinese and Western dogs. The genotyping data of 170K SNP chips in Western dogs were downloaded from the LUPA (a European initiative of canine genome project) database. Chinese indigenous dogs had lower LD and shorter accumulative runs of homozygosity (ROH) in the genome. The genetic distances between individuals within each Chinese breed were larger than those within Western breeds. Chinese indigenous and Western dog breeds were clearly differentiated into two separate clades revealed by the PCA and NJ-tree. We found evidence for historical introgression of Western dogs into Chinese Kazakhstan shepherd and Mongolia Xi dogs. We suggested that Greenland sledge dog, Papillon, and European Eurasier have Chinese dog lineages. Selection sweep analysis identified genome-wide selection signatures of each Chinese breed and three breed groups. We highlighted several genes including *EPAS1* and *DNAH9* that show signatures of natural selection in Qinghai-Tibetan plateau dogs and are likely important for genetic adaptation to high altitude. Comparison of our findings with previous reports suggested *RBP7*, *NMNAT1*, *SLC2A5*, and *H6PD* that exhibit signatures of natural selection in Chinese mountain hounds as promising candidate genes for the traits of endurance and night vision, and *NOL8*, *KRT9*, *RORB*, and *CAMTA1* that show signals of selection in Xi dogs might be candidate genes influencing dog running speed. The results about genomic and population structures, and selection signatures of Chinese dog breeds reinforce the conclusion that Chinese indigenous dogs with great variations of phenotypes are important resources for identifying genes responsible for complex traits.

## Introduction

Of all the domesticated animals, dogs (*Canis familiaris*), are one of the most popular species. Archaeological evidence suggests that the dog was the first domesticated animal (Clutton-Brock, 1995). Humans domesticated dogs in two stages. The first stage occurred over 15,000 years ago. The dogs in this stage came from a population of wolf-like progenitors. The second stage began only in the last few hundred years; in this stage, human selection began and specific dog breeds appeared (Lindblad-Toh et al., 2005; Wayne and Ostrander, 2007; Wang et al., 2014b). There are many speculations about what wolf species was the origin of domestic dog. Scientists from Switzerland and China claim that dogs originated from the Asian Grey Wolf in Southeast Asia over 33,000 years ago (Leonard et al., 2002; Savolainen et al., 2002). Approximately 15,000 years ago, a portion of ancestral dogs migrated from East Asia to the Middle East, Africa, and Europe, with arrival in Europe dated at about 10,000 years ago (Wang et al., 2016). Thalmann et al. (2013) compared the ancient mitochondrial DNA (mDNA) of 18 fossils from canids against the mDNA of 49 modern wolves and 77 modern dogs. The authors pinpointed Europe as the major nexus of dog domestication. Shannon et al. (Shannon et al., 2015) published an alternative origin story and suggested that domestic dogs originated from central Asia. This conclusion was based on autosomal, mitochondrial, and Y chromosome diversity data from 4,676 purebred dogs (161 breeds, 549 cities, and 38 countries). Frantz et al. (2016) reported that dogs may have been domesticated independently in Eastern and Western Eurasia from distinct wolf populations. Therefore, the geographic and temporal origins of domestic dogs remain controversial.

In China, there are nearly three dozen indigenous dog breeds. Based on geographical distribution, dogs indigenous to China can be classified into three groups: Mountain hounds, Qinghai-Tibet plateau dogs, and plain dogs. The native domestic breeds are highly adapted to local environmental conditions. People generally sort Chinese indigenous dogs into categories based on how they are used (e.g., hounds, shepherd dogs, guard dogs, pets, etc.); artificial selection for specific phenotypes is an important driving force behind the diversity of Chinese dog breeds. Therefore, Chinese indigenous dogs are important genetic resources for studying the formation of specific breeds and for identifying genes responsible for current phenotypic variations.

Recent years, many more studies about the domestication and evolution of Chinese dogs appeared (Li et al., 2014; Wang et al., 2014a; Hao et al., 2016; Wang et al., 2016). Several causative genes responsible for phenotypic variations of Chinese indigenous dogs have also been reported. For instance, *EPAS1* and *HBB* were identified as the responsible genes for hypoxic adaptation of Tibetan dogs (Wang et al., 2014a). However, to the best of our knowledge, there is a lack of research about the genetic diversity, genomic structure, evolutionary relationships, and selection signatures in many Chinese indigenous dog breeds. Therefore, this study aims to investigate the genetic variability of the Chinese dog population and compare that to Western dogs. Through these comparisons, we hope to acquire more information about the evolution of the domestic dogs.

## Material and Methods

### Animals

**Figure 1:**
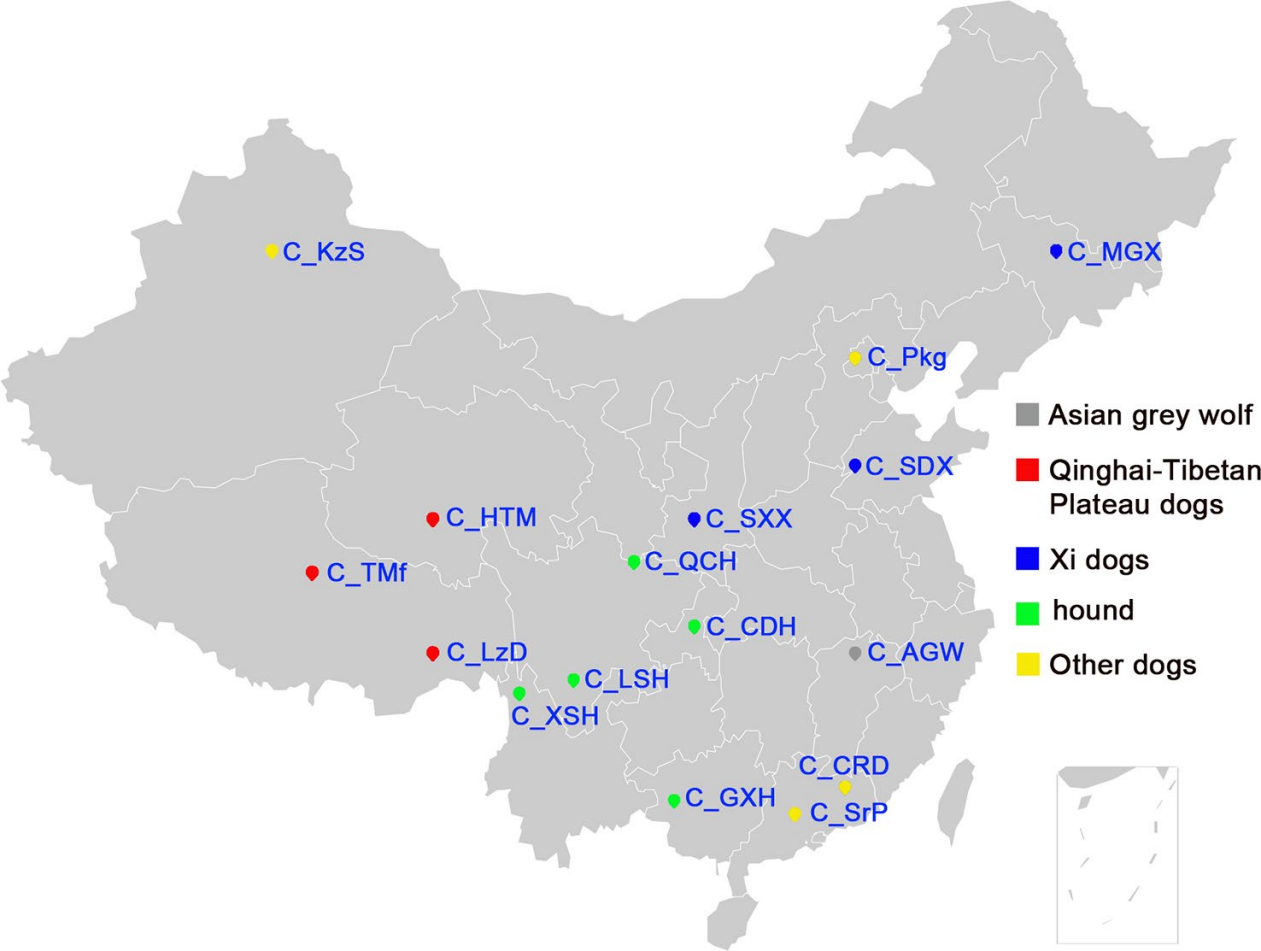
The geographic locations of Chinese indigenous dogs. The geographical distribution of sampled Chinese indigenous dogs, the full name of each breed is listed in **Table 1**.

We genotyped 173 dogs, including 157 dogs from 15 Chinese indigenous dog breeds, 10 Rottweiler and 3 Papillons as controls, and 3 Asian wolves (**Table 1**). **Figure 1** shows the geographical distribution of the 15 Chinese dog breeds. Asian wolf samples were collected from the Nanchang Zoo. The samples of 10 Rottweiler and 3 Papillons were collected from service dog station in Nanchang, Jiangxi Province. We checked the pedigree records carefully before sampling for each breed to avoid the closely related samples. Trained veterinarians collected all blood samples according to the Standard of Public Safety of China. Genomic DNA was extracted using SE Blood DNA Kit (OMEGA, USA) according to the manufacturer’s instructions. The quality of genomic DNA was assessed by Nanodrop-1000 (Thermo Scientific, USA) and 0.8% agarose gel electrophoresis. All DNA samples were diluted to 40 ng/µl for genotyping. In addition, the 170K SNP genotyping data from the 458 Western dogs was downloaded from the dataset generated by the LUPA consortium (Vaysse et al., 2011) and used to compare the genetic diversity between Chinese and Western dogs.

**Table 1:** Genetic parameters and linkage disequilibrium extent of Chinese and Western dog populations.

Population	Origin	No.	Abb.	N_snp_	Indices of Genetics Diversity				r^2^0.3 (kb)	inbreeding coefficient	Genotyping data from
					P_N_	A_R_	H_E_	H_O_		F	
Asian grey wolf	China	3	C_AGW	43,966	0.48	1.48	0.19	0.28	–	0.35	*
Chuandong hound	Chongqing	12	C_CDH	64,219	0.87	1.88	0.29	0.30	45.51	0.31	*
Chinese country dog	Guangdong	9	C_CRD	81,323	0.96	1.96	0.36	0.36	22.72	0.17	*
Guangxi hound	Guangxi	12	C_GXH	73,850	0.94	1.95	0.34	0.34	21.51	0.21	*
Kazakhstan shepherd dog	Xinjiang	4	C_KzS	84,325	0.92	1.92	0.36	0.39	–	0.09	*
Liangshan hound	Sichuan	12	C_LSH	67,104	0.90	1.91	0.31	0.32	46.35	0.26	*
Linzhi dog	Tibet	12	C_LzD	80,221	0.97	1.98	0.37	0.37	26.37	0.14	*
Mongolia Xi dog	Inner Mongolia	12	C_MGX	82,543	0.98	1.99	0.38	0.40	27.16	0.08	*
Pekingese	Beijing	3	C_Pkg	75,408	0.82	1.82	0.33	0.36	–	0.17	*
Qingchuan hound	Sichuan	12	C_QCH	75,664	0.95	1.96	0.35	0.35	27.54	0.20	*
Shandong Xi dog	Shandong	12	C_SDX	77,731	0.96	1.97	0.36	0.38	35.45	0.14	*
SharPei	Guangdong	21	C_SrP	74,964	0.97	1.98	0.35	0.34	46.33	0.22	*
Shanxi Xi dog	Shanxi	12	C_SXX	65,413	0.86	1.87	0.30	0.31	77.79	0.28	*
Hequ Tibetan mastiff	Gansu	7	C_HTM	73,909	0.90	1.90	0.34	0.39	53.09	0.10	*
Tibetan mastiff	Tibet	15	C_TMf	84,308	0.98	1.99	0.38	0.36	23.23	0.17	*
Xiasi hound	Guizhou	12	C_XSH	74,280	0.95	1.96	0.34	0.34	18.95	0.21	*
Belgian Tervuren	Belgium	12	W_BeT	69,429	0.89	1.89	0.32	0.33	89.00	0.24	**
Beagle	England	10	W_Bgl	73,144	0.89	1.89	0.31	0.32	72.66	0.27	**
Bernese Mountain dog	Switzerland	12	W_BMD	59,370	0.83	1.84	0.28	0.29	150.8	0.35	**
Border Collie	England	16	W_BoC	73,276	0.95	1.98	0.34	0.35	66.91	0.19	**
Border Terrier	England	25	W_BoT	63,539	0.85	1.85	0.28	0.28	168.5	0.37	**
Brittany Spaniel	France	12	W_BrS	77,530	0.96	1.97	0.36	0.35	50.71	0.20	**
Papillon	France	3	W_Pap	74,624	0.81	1.81	0.33	0.41	–	0.05	*
Cocker Spaniel	England	14	W_CoS	76,410	0.95	1.95	0.34	0.32	71.99	0.26	**
Dachshund	Germany	12	W_Dac	79,874	0.97	1.97	0.37	0.35	44.15	0.19	**
Doberman Pinscher	Germany	25	W_Dob	60,009	0.88	1.93	0.27	0.25	205.2	0.42	**
English Bulldog	England	13	W_EBD	60,468	0.84	1.85	0.27	0.25	138.4	0.42	**
Elkhound	England	12	W_Elk	77,561	0.95	1.96	0.36	0.35	49.50	0.21	**
English Setter	England	12	W_ESt	71,106	0.92	1.93	0.33	0.33	73.24	0.24	**
Eurasier	Europe and Asia	12	W_Eur	72,411	0.93	1.93	0.33	0.35	65.84	0.21	**
Finnish Spitz	Finland	12	W_FSp	65,032	0.86	1.87	0.30	0.31	100.9	0.30	**
Gordon Setter	England	25	W_GoS	80,738	0.98	2.03	0.36	0.34	53.52	0.22	**
Golden Retriever	Canada	14	W_GRe	76,318	0.96	1.96	0.34	0.33	60.09	0.25	**
Greyhound	Italy	11	W_Gry	63,515	0.87	1.87	0.30	0.27	101.9	0.37	**
German Shepherd	Germany	12	W_GSh	61,418	0.85	1.85	0.28	0.28	123.9	0.36	**
Greenland sledge dog	Canada	12	W_GSl	53,982	0.82	1.82	0.25	0.25	90.36	0.43	**
Irish Wolfhound	Ireland	11	W_IrW	54,601	0.74	1.74	0.26	0.26	225.6	0.41	**
Jack Russell Terrier	England	12	W_JRT	83,934	0.99	1.99	0.39	0.40	37.48	0.10	**
Labrador Retriever	Canada	14	W_LRe	79,979	0.96	1.96	0.36	0.34	59.63	0.21	**
Newfoundland dog	Canada	25	W_NFd	76,133	0.95	2.00	0.34	0.33	77.60	0.25	**
Nova Scotia Duck Tolling Retriever	Canada	23	W_NSD	70,421	0.91	1.91	0.32	0.32	101.6	0.26	**
Rottweiler	Germany	22	W_Rtw	64,439	0.89	1.90	0.30	0.29	132.2	0.33	***
Schipperke	Belgium	25	W_Sci	76,758	0.95	2.06	0.34	0.35	73.33	0.21	**
Standard Poodle	France	12	W_StP	76,733	0.95	1.95	0.35	0.36	69.01	0.18	**
Terrier Yorkshire	England	12	W_TYo	77,523	0.96	1.96	0.36	0.36	51.77	0.17	**
Weimaraner	Germany	26	W_Wei	59,404	0.88	1.88	0.28	0.27	172.1	0.37	**
Chinese indigenous dogs	China	170	C_dog	108,242					8.49	0.19	
Western dogs	Western	458	W_dog	117,869					15.07	0.27	
All dogs		628	All_dog	119,427							

No. indicates the number of individual for each breed; Abb. represents the breed abbreviation that used in this article; N_SNP_, the number of SNPs with MAF >0.1; P_N_, the proportion of the SNPs which displayed polymorphism in each dog breed in the all 131,927 SNPs passed the quality control; A_R_, allelic richness; H_E_, expected heterozygosity; H_O_, observed heterozygosity; r^2^_0.3_ were calculated between all pairs of SNPs with MAF ≥5% and <10% missing data in each population.

*Genotyping data from this study, and **from Vaysse et al. (2011). ***10 samples of Rottweiler were collected from service dog station in Nanchang, Jiangxi province, and genotyped in this study.

### Genotyping and Quality Control

All 173 samples were genotyped using Canine 170K SNP BeadChips (containing 173,662 SNPs) on an iScan System (Illumina, USA). The examined SNPs in the dataset downloaded was slightly different from our data because SNP positions were annotated using different reference genome assemblies (v3.0 vs. v2.0). To deal with these differences, the 120-bp flanking sequences for all SNPs in the downloaded dataset were extracted. And then, these short sequences were mapped to the dog reference genome assembly (v3.0) to identify the common SNPs with positional information (Kent, 2002). A total of 173,392 SNPs were mapped to the reference genome assembly (v3.0). After quality control, a common subset data (151,057 SNP markers) was obtained for further analysis using PLINK (v1.9) (Purcell et al., 2007). The SNPs with minor allele frequency (MAF) <0.01, and a call rate < 90% were further filtered. The individuals with call rates < 95% were also excluded from further analysis. Finally, 131,927 SNPs and 628 individuals were retained for further study.

### Genetic Diversity and Population Structure Analysis

To avoid the effect of SNP polymorphism (e.g., rare SNPs) on estimating genetic diversity of dog breeds, we used a subset of 119,427 SNPs with MAF >0.1 in both Chinese and Western dogs for calculating the parameters of genetic variability. Variability parameters of the study were the Proportion of polymorphic markers (P_N_), allelic richness (A_R_), expected heterozygosity (H_E_), observed heterozygosity (H_O_), and inbreeding coefficient (F). A_R_ was estimated with ADZE software (v1.0) (Szpiech et al., 2008). P_N_, H_E_, H_O,_ and F were calculated with PLINK (v1.9) (Purcell et al., 2007) under the default settings. All 131,927 SNPs with MAF >0.01 and call rate >90% were used to estimate the genetic distance (D_st_) between populations using PLINK (v1.9). Genetic distance between all pairwise combinations of individuals was calculated as 1-Dst.

To avoid artifacts due to linkage disequilibrium (LD), we used a subset of 95,503 informative SNPs with MAF >0.05, call rate >90%, and pairwise genotype *r^2^* <0.5 for conducting principal component analysis (PCA) and calculating IBS distance matrix values among Chinese dogs (170 individuals) with the argument *cluster-distance-matrix* under default parameters. Furthermore, under the same criteria, a subset of 97,017 informative SNPs was used to perform PCA and population structure analysis between Chinese and Western dogs (628 individuals). PCA was carried out using --*pca* command in GCTA software (Yang et al., 2011). The IBS distance matrix was converted to Neighbor-joining (NJ) (Felsenstein, 1989) relationship trees using the Neighbor method in the PHYLIP package (v3.69) (Plotree and Plotgram, 1989). Phylogenetic trees were constructed by FigTree (v1.4.2). For population structure analysis, we randomly selected 12 individuals for each breed and estimated lineage component using ADMIXTURE software with the unsupervised fashion (Alexander et al., 2009) and default parameters. The results were plotted using R software.

### Linkage Disequilibrium Decay Assay

The autosomal SNPs with MAF ≥ 5% and call rate ≥90% in each dog population were used to analyze LD between SNPs. The genotype correlation coefficient (*r*^2^) was used to measure the pairwise LD. *r*^2^ values were calculated by PLINK (v1.9) with the command *-- r2 --ld-window-kb 500 --ld-window-r2 0* (Ai et al., 2013). To improve the accuracy of the estimation, and facilitate the comparison of LDs among dog breeds, we removed breeds with sample size <5, including the Kazakhstan shepherd (four samples) and the Pekingese (three samples). Sample sizes for all breeds were kept at 12 individuals, except for the Hequ Tibetan mastiff and Chinese country dog, in which only seven and nine dogs, respectively, were genotyped. We compared the LD decay between Chinese dogs (157 dogs from Chinese indigenous breeds) and a random subset of Western breeds (n = 157).

### Identification of Runs of Homozygosity

Runs of homozygosity (ROH) were identified by PLINK (v1.9) with the command *--homozyg-snp* and *--homozyg-kb*. The program aligned a moving window of 50 SNPs across the genome to detect long contiguous ROHs genotypes in each population. ROH could be underestimated if there is a genotyping error or missing genotype that occurs in an otherwise unbroken homozygous segment. The program was set to allow one heterozygous and five missing calls per window. This analysis was performed on a subset of 115,014 SNPs that were pruned for strong LD (*r*^2^ ≥ 0.8). Because strong LDs up to 100 kb are common throughout the dog genome, and short tracts of homozygosity are very prevalent, the minimum length for an ROH was set at 200 kb.

### Detection of Genetic Introgression

To infer the genetic differentiation and admixture among populations, we utilized TreeMix (v1.13) to construct a maximum-likelihood tree (Pickrell and Pritchard, 2012). To account for the fact that nearby SNP are not independent, and considering the SNP density (∼1SNP/20K) obtained in this study and LD extent of modern dog breeds, we grouped 300 SNPs together in windows that far exceeds the known extent of LD in dogs, and set Asian Grey Wolf (C_AGW) as the outgroup population in the analysis. The SNPs with MAF ≤0.05, a call rate ≤90%, and the SNPs located on sex chromosomes were excluded from analysis. We used the data set with 128,034 SNPs to estimate genetic differentiation and admixture among 46 populations under 0–5 migration events via TreeMix (v1.13). In addition, a *D*-statistics [also called 4-taxon ABBA-BABA test, *D* (P1, P2, P3, Outgroup)] was performed using Admixtools (v5.1) (Loh et al., 2013).

### Estimation of Population Genetic Differentiation

We calculated unbiased estimates of pairwise F_ST_ using a previously described method (Akey et al., 2002) with the SNP data set passing quality control (131,927). The formula was defined as Eq. (1):

(1a) $$F_{st}=\frac{MSP-MSG}{MSP+({\text{n}}_{c}-1)MSG}$$

(1b) $$MSG=\frac{1}{\Sigma_{i=1}^{S}\;n_{i}}\Sigma_{i}^{S}n_{i}P_{Ai}(1-P_{Ai})$$

(1c) $$MSP=\frac{1}{s-1}\Sigma_{i}^{s}\;n_{i}{\left(P_{Ai}-\bar{P_{A}}\right)}^{2}$$

(1d) $$n_{c}=\frac{1}{s-1}\sum_{i=1}^{S}n_{i}-\frac{\Sigma_{i}\;n_{i}^{2}}{\Sigma_{i}\;n_{i}}$$

(1e) $$\bar{P_{A}}=\frac{n_{i}P_{Ai}}{\Sigma_{i}\;n_{i}},$$

where *MSG* and *MSP* represent observed mean-squared error of the frequency of allele A within and between populations, respectively. $\bar{P_{A}}$ is a weighted average of *P_A_* across populations. *n_c_* is the average sample size across populations; it incorporates and corrects for the variance in sample size over populations. *P_Ai_* denotes the frequency of allele A in the *i*-th population (where *i* = 1, …, *s*). The *i*-th population size is shown with *n_i_*. The *F_ST_* values ranged from 0 to 1. A zero value indicates no population structuring or subdivision (complete panmixis), and 1 implies that all genetic variation is explained by the population structure. Meaningless negative *F_ST_* values were set to 0. Pairwise *F_ST_* values between breeds were calculated by Genepop software (v4.5.1) (Rousset, 2008).

### Detection of Selection Signature

We calculated the statistical *d_i_* values to identify SNPs that suggest selection signatures in the 13 Chinese indigenous breeds (because both C_HTM and C_TMf belong to Tibetan mastiff, we merged them together in selection sweep analysis. C_KzS and C_Pkg were removed for small sample size). To detect selection signatures associated with specific phenotypes (high-altitude adaption, running speed and hunting ability), we performed the *d_i_* statistics on three contrast models: plateau dogs (Tibetan) vs. non-plateau dogs, fast running dogs (Xi dogs) vs. non-fast running dogs and mountain hounds against non-mountain hounds (**Supplementary Table S1**). The *d_i_* estimate is based on the level of population differentiation. It detects lineage-specific selection events and determines recent or preexisting selection signatures (Akey et al., 2010). The locus-specific divergence in allele frequencies for each breed, within 200-kb windows across the 38 autosomes, was calculated using the *d_i_* statistics. For each SNP, we calculated *d_i_* values with Eq. (2):

(2) $$d_{i}=\Sigma_{j\neq i}\frac{F_{ST}^{ij}-E[F_{ST}^{ij}]}{sd\;[F_{ST}^{ij}]}$$

$E[F_{ST}^{ij}]$ and $sd[F_{ST}^{ij}]$ represent the expected value and standard deviation of *F_ST_* between breeds *i* and *j* estimated from all 131,927 SNPs. The mean of *d_i_* values across SNPs was taken for each of the 200-kb non-overlapping sliding windows for autosomal SNPs. The windows containing fewer than six SNPs were discarded. The average number of SNPs per window was 12.5. Only those windows fell into the upper 99.5th percentile of the empirical distribution were considered as the candidate selection regions. We further analyzed the selection signature of three specific dog groups that we categorized according to their geographical distribution (high-altitude adaption), purpose of utilization (mountain hounds), and special ability (fast running speed in hunting). The population clustering result obtained in this study was also considered in the determination of these Chinese dog groups (**Supplementary Table S1**). Similar to previous descriptions, we estimated *d_i_* values for each group to detect the significant selection signature regions.

To further identify some more confident signals, a haplotype-based analysis was also used to detect signatures of selection under 200-kb windows within breeds and between breed groups by the Selscan software, in which integrated haplotype score (iHS) was calculated to track the decay of haplotype homozygosity for both the ancestral and derived haplotypes extending from a query site by using Extended Haplotype Homozygosity (EHH) (Szpiech and Hernandez, 2014).

### Annotation of Candidate Genes Underlying Selection

We used an online tool, VEP (Variant Effect Predictor), to predict the potential effects of the SNPs; the tool returned selection signatures on known gene functions. We identified candidate genes located within each selected region using the reference genome assembly (v3.0). Genecards (Stelzer et al., 2016) and NCBI annotated the gene functions. GO function annotated the functional enrichments of candidate genes underlying selection.

## Results

### Data Characteristics

We genotyped 173,662 SNPs with the current Illumina Canine 170K Beadchip (v3.0) in a panel of 157 unrelated dogs that were from 15 phenotypically and genetically diverse breeds and three Asian wolves (**Table 1**). In addition, we compared the population structure and genetic diversity of Chinese and Western dogs by assessing the genotyping data of 173,404 SNPs in the 458 Western dogs’ data downloaded from the LUPA database. After quality control, 131,927 SNPs including 3,895 SNPs on chromosome X (CFA X), were used for further statistical analyses. The numbers of polymorphic SNPs (MAF ≥0.1) in each dog population are shown in **Table 1**. In general, Chinese indigenous dogs had a higher average number of polymorphic SNPs than Western dogs (75,684 vs. 70,323). In Chinese dog breeds, C_KzS (Kazakhstan shepherd dogs) had the highest number of polymorphic SNPs (84,325), while C_CDH (Chuandong hunter) possessed the fewest number of polymorphic SNPs (64,219). In Western dog populations, the breeds with the greatest and fewest number of polymorphic SNPs were W_JRT (Jack Russell Terrier; 83,934) and W_GSl (Greenland sledge dog; 53,982), respectively.

### Genetic Diversity Across Chinese and Western Dogs

We performed independent calculations of P_N_, A_R_, H_E_, and H_O_ for each of 15 Chinese breeds, 30 Western breeds, and Asian grey wolves (**Table 1**). Overall, Chinese and Western dogs had comparable proportion of polymorphic SNPs (P_N_ range: dogs, 0.82 to 0.98; Western dogs, 0.74 to 0.99). In Chinese dog breeds, C_MGX (Mongolia Xi) and C_TMf (Tibetan mastiff) displayed the highest genetic diversity as measured by allelic richness (A_R_ = 1.99), expected heterozygosity (H_E_ = 0.38), and observed heterozygosity (H_O_ = 0.4 and 0.36). However, C_CDH (Chuandong hound) showed the lowest SNP variability (P_N_ = 0.87, A_R_ = 1.88, H_E_ = 0.29, H_O_ = 0.30). In Western dogs, the highest genetic diversity was identified in W_JRT (Jack Russell Terrier) (P_N_ = 0.99, A_R_ = 1.99, H_E_ = 0.39, H_O_ = 0.40), while the lowest diversity was observed in W_IrW (Irish Wolfhound) (P_N_ = 0.74, A_R_ = 1.74, H_E_ = 0.26, H_O_ = 0.26).

### Genetic Distance Within and Between Populations

**Figure 2:**
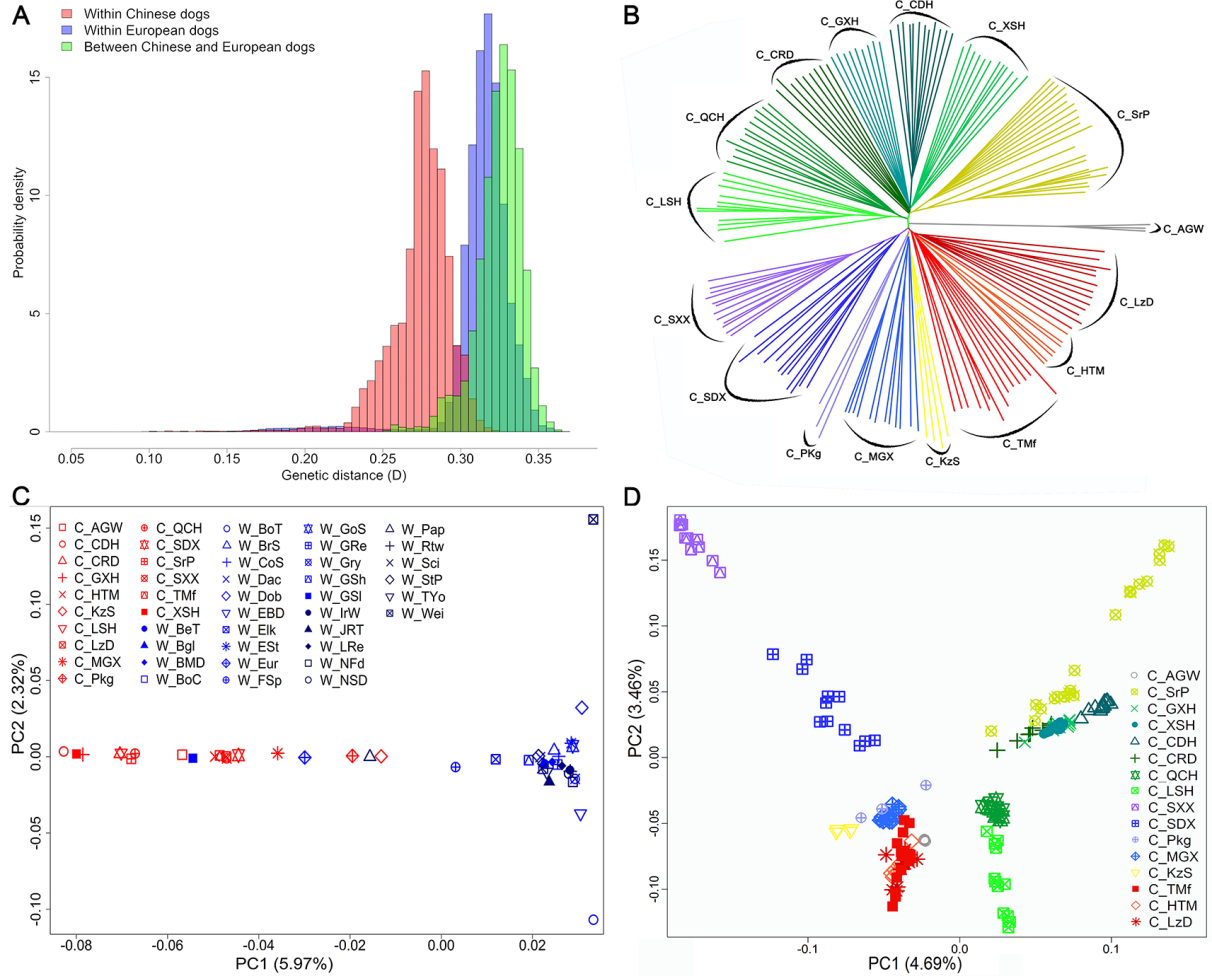
Genetic diversity, and population structures within and between Chinese and Western dogs. **(A)** The genetic distances between pairs of animals. Light red bars, light blue bars, and light green bars represent genetic distance within Chinese dogs, within Western dogs, and between Chinese and Western dogs, respectively. **(B)** The neighbor-joining (NJ) tree of the exampled Chinese indigenous dog populations based on genome-wide allele sharing. **(C)** Population structures of Chinese and Western dogs revealed by principal component analysis. **(D)** Principal component analysis revealed the population structures of Chinese dogs. Each colorful and shaped point represents a comprehensive value of one breed. The full name of each breed is listed in **Table 1**.

The average genetic distance across Chinese dog breeds was 0.27 ± 0.02, across Western dog breeds, it was 0.31 ± 0.02, and between Chinese and Western dogs it was 0.33 ± 0.01 (**Figure 2A**). Genetic distance across individuals within each breed ranged from 0.20 ± 0.01 (C_CDH) to 0.27 ± 0.01 (C_Pkg, Pekingese) in Chinese dog breeds, and from 0.17 ± 0.01 (Greenland sledge dog, W_GSl) to 0.27 ± 0.01 (W_JRT) in Western dog breeds (**Supplementary Figure S1**). Interestingly, the average genetic distance of all the tested Chinese dog breeds (0.24 ± 0.03) was larger than that of Western dog breeds (0.22 ± 0.02), suggesting Chinese dogs have either a longer history than Western dogs or had not experienced the stringent breeding programs and periodic population bottlenecks.

To investigate topological relationships between Chinese and Western dogs and among Chinese dog breeds, we constructed an NJ-tree based on genome-wide allele sharing. Chinese and Western dogs were clearly clustered into two separate clades; however, three Western dogs (W_Pap (Papillon), W_Eur (Eurasier), and W_GSl (Greenland sledge dog)) were clustered into Chinese populations In addition, two Chinese dog breeds (C_KzS and C_MGX) were grouped into clades distinct from the major Chinese clade (**Supplementary Figure S2**). Interestingly, C_AGW (Asian grey wolf) and W_GSl formed two close clades that were located between the clades of plateau dog breeds (C_HTM, C_TMf, and C_LzD) and Southwest dog breeds including Chinese hounds (C_CDH, C_LSH, C_QCH, and so on), rural dog (C_CRD), and Shar-Pei (C_SrP). Ten Rottweilers genotyped in this study were perfectly clustered in the clade with the Rottweilers from the LUPA dataset. We observed four notable features in the NJ-tree among Chinese breeds. First, all individuals within each breed were clustered together. Second, the relationship between individuals within each breed was more distant than in Western breeds. Third, the 15 Chinese breeds were obviously divided into two main clusters. One cluster encompassed seven breeds from Southwest China and the other cluster contained dogs from Northwest China. Finally, we were able to divide each main cluster into 2∼3 sub-clusters, which represented different geographic subpopulations (**Figure 2B**).

### Population Structure of Chinese and Western Dogs

We analyzed the population structures of all 46 Chinese and Western dog populations using PCA with the filtered genotype data of 97,017 SNPs (described in the *Methods*). The results clearly distinguished Chinese dogs from Western dogs on the first two eigenvector axes. The PC1, which counts for 5.97% component, indicated a genetic difference between Chinese and Western dogs (**Figure 2C**). We focused on the population structure of 15 Chinese dog populations and found that all dogs from high-attitude regions (Qinghai-Tibet plateau), including C_HTM, C_TMf, and C_LzD, were clustered together. However, other Chinese breeds were separately distributed (**Figure 2D**). Interesting, the PC1 (4.69%) and PC2 (3.46%) formed a similar structure with NJ-tree findings (**Figures 2B, D**).

**Figure 3:**
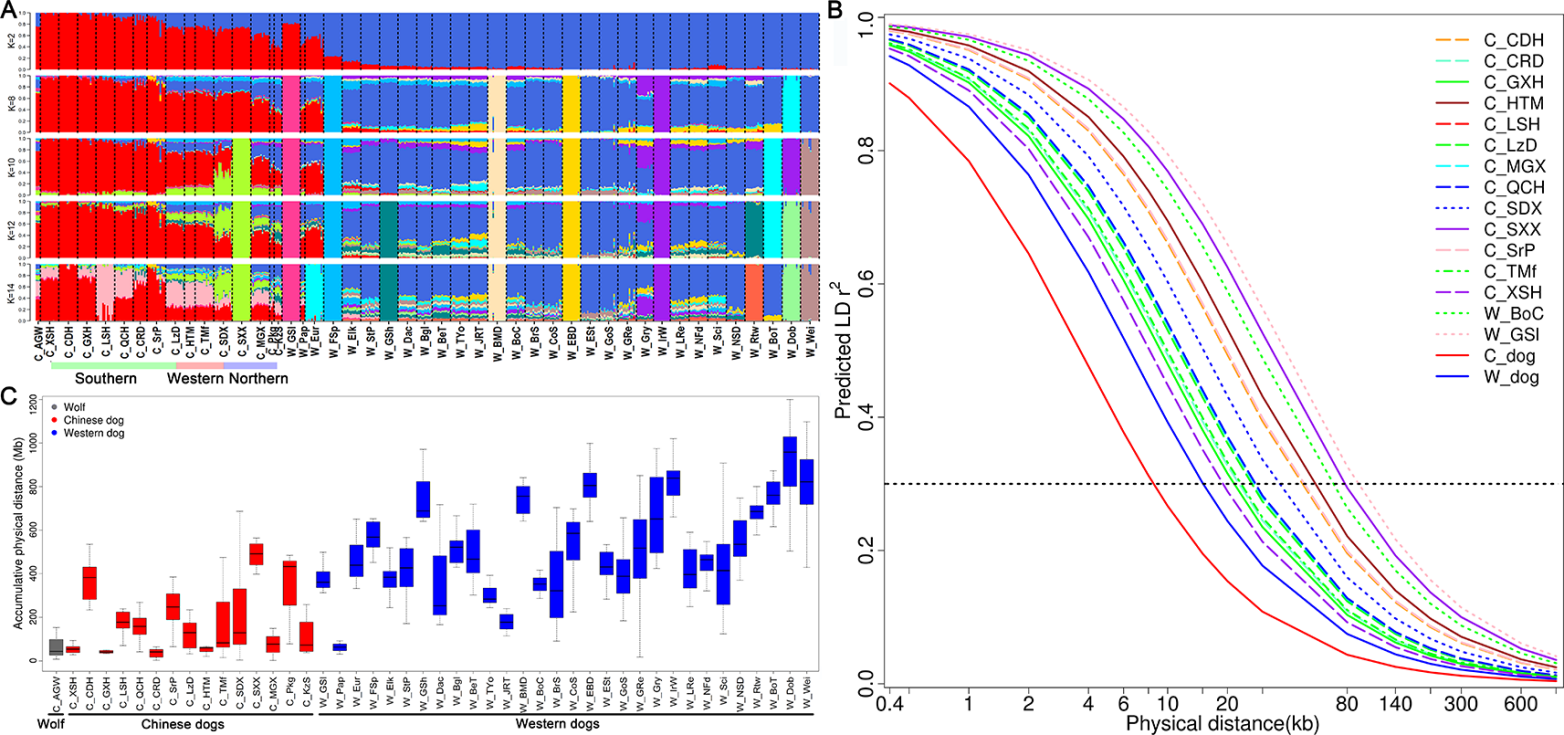
Admixture, runs of homozygosity, and genome-wide linkage disequilibrium (LD) of Chinese dogs compared with Western dogs. **(A)** Admixture analysis in Chinese indigenous dog populations compared with Western dogs (from K = 2 to K = 6). Red bars represent Chinese dog breeds and blue bars indicate Western dog breeds (K = 2). **(B)** Decline in genome-wide linkage disequilibrium (predicted *r*^2^) across and within breeds. **(C)** Accumulative physical length of runs of homozygosity (ROH) in Chinese and Western dog populations.

To quantify population structure and admixture patterns of the tested populations, we calculated Q values using ADMIXTURE software with a subset of SNP data (see Methods). Chinese indigenous dogs were completely segregated from Western dogs (*K* = 2). This was followed by W_EBD (English Bulldog) and W_BMD (Bernese Mountain dog), W_Dob (Doberman Pinscher), W_IrW (Irish Wolfhound), W_FSp (Finnish Spitz), and W_GSl (Greenland sledge dog) (*K* = 8), until C_SXX (Shanxi Xi dog) (*K* = 10) and C_LSH (Liangshan hound) (*K* = 14) segregated (**Figure 3A**), indicating a major difference between Chinese and Western dogs and a distinct lineage of Western dogs. Interestingly, we observed a significant relationship amongst W_GSl, W_Pap, and W_Eur with Chinese dogs, implying that the Greenland sledge (W_GSI) dog likely migrated from China or admixed with Chinese breeds (**Figure 3A**).

### Inbreeding and Admixture in Dog Breeds

To improve the accuracy of data analyzed in this study, we used the same sample size for estimating *r^2^* values of each population (see *Methods*). We calculated the *r*^2^ values of all pairs of autosomal SNPs with MAF >5% and call rate >90% within each population. The numbers of SNPs used for *r*^2^ estimates ranged from 82,485 to 108,509 in Chinese dog populations, and from 70,436 to 107,280 in Western dog populations. We set the *r^2^* threshold to 0.3 (*r^2^*_0.3_) for pairwise comparisons of LD extent patterns. The LD values (*r^2^*_0.3_) ranged from 18.95 Kb (C_XSH, Xiasi hound) to 77.79 Kb (C_SXX, Shanxi Xi dog) in Chinese populations (**Table 1** and **Figure 3B**) and from 37.48 Kb (W_JRT) to 225.60 Kb (W_IrW) in Western dogs (**Table 1** and **Supplementary Figure S3**). Notably, the extent of LD within inter-population across Chinese dogs (*r^2^*_0.3_ = 8.49 Kb) is much lower than that across Western dogs (*r^2^*_0.3_ = 15.07 kb) (**Table 1** and **Supplementary Figure 3B**).

To evaluate the effect of inbreeding on the dog genome, we assessed the genome-wide autozygosities as ROH. In general, Western dogs had a higher fraction of ROH than Chinese dog populations (**Figure 3C**). Western dogs showed both higher LD extent and larger accumulative ROH, which indicates recent inbreeding in Western breeds. Similarly, Western dogs have a higher inbreeding coefficient than Chinese dogs (0.27 vs. 0.18, **Table 1**). It is most likely the experience of the stringent breeding programs and periodic population bottlenecks that causes inbreeding and larger LD during the formation of modern Western dog breeds (Lindblad-Toh et al., 2005). In Chinese dog populations, C_SXX had the highest ROH value, inbreeding coefficient (0.28), and the largest LD extent (*r^2^*_0.3_ = 77.79 Kb), suggesting recent inbreeding or bottlenecks in this breed. Hequ Tibetan mastiff (C_HTM) exhibited the shortest ROH and the smallest inbreeding coefficient (0.1), but the largest LD extent (*r^2^*_0.3_ = 53.09 Kb) in the genome analyses (**Figures 3B, C**). This is likely a result of recent admixture.

To estimate the history and pattern of introgression in Chinese and Western dogs, we used TreeMix to estimate an admixture tree with 0-7 migration events (**Supplementary Figures S4**–**S7**). We inferred introgression events from various groups: 1) Chinese dogs to Western dogs (W_GSl, W_Eur, and W_Pap), 2) Chinese hound dogs (C_CDH, C_GXH, and C_XSH) to Chinese Xi dogs (C_SXX and C_SDX), 3) Western dogs to Chinese dogs (C_MGX and C_KzS). These signals of genetic introgression were supported by *D*-statistics analyses (ABBA-BABA tests) (**Supplementary Table S2**).

### Population Differentiation Within Chinese Dogs

To reduce the deviation of *F_st_* estimates caused by the different sample size of each breed, we selected Chinese dog populations that had more than seven individuals. Pairwise *F_st_* values were estimated for all autosomal informative SNPs (**Supplementary Figure S8**). As expected, C_AGW displayed the largest divergence from Chinese indigenous dogs (*F_st_* = 0.272 ± 0.034). Chinese rural dogs (C_CRD) showed the lowest population differentiation (*F_st_* = 0.086) compared with the other Chinese dogs (*F_st_* = 0.128). In addition, population differentiation between C_XSH and C_CRD was the lowest with an *F_st_* value of 0.009. However, C_SXX exhibited the highest divergence with C_AGW having an *F_st_* value of 0.350.

### Selection Signature in Individual Chinese Dog Breeds

A genome-wide scan for selection signatures in 13 Chinese breeds and Asian wolves was performed by *d_i_* statistics and iHS. The *d_i_* values were calculated for autosomal SNPs in 200-kb windows as described by Wei et al. (2015). We evaluated a total of 10,989 windows involving 117,492 SNPs. We defined candidate selection regions that fell into the upper 99.5th percentile of the empirical distribution. In total, 650 windows falling into 528 chromosomal regions were detected selection signatures in all 13 tested populations (50 selection sweep windows for each breed in average) (**Supplementary Table S3**). C_GXH (Guangxi hound) and C_HTM (Hequ Tibetan mastiff) had the largest number (46) of selection regions, while C_SrP (Chinese Sharpei) had the fewest number (30) of selection regions. The maximal *d_i_* statistic value was identified at CFA 6: 37.6 –38.2 Mb in C_SrP (**Supplementary Figure S9** and **Table S3**). To further verify the selection signals detected by *d_i_* statistics, we repeated the selection signature analysis by using iHS method that based on extended haplotype homozygosity in each population. As a result, 27 selection signatures were repeated each other between *d_i_* and iHS analysis (**Supplementary Figures S9**–**S11** and **Table S3**).

We performed a functional annotation for all genes showing signatures of selection in each of Chinese dog breeds with GO function terms and KEGG pathways. The genes identified in different dog breeds were enriched in the different GO and KEGG terms. For examples, the genes identified in C_SDX and C_SXX were enriched for the GO function term “response to interleukin-15” and the KEGG pathway “estrogen signaling” (**Supplementary Table S4**); The genes that showed selection signals in C_TMf were enriched for the “multicellular organism growth processes”. As we have well known, Tibetan mastiffs have large body size. The GO function terms of “oxidoreductase activity processes” and “nuclear envelope organization processes” were enriched by the genes identified in C_QCH and C_SrP, respectively (**Supplementary Table S4**). However, some of these GO terms are very general. Whether it implies some kind of relationship between the selection sweep genes and dog phenotypes remains to be further studied.

### Signatures of Selection in Plateau Dogs, Xi Dogs, and Mountain Hounds

**Figure 4:**
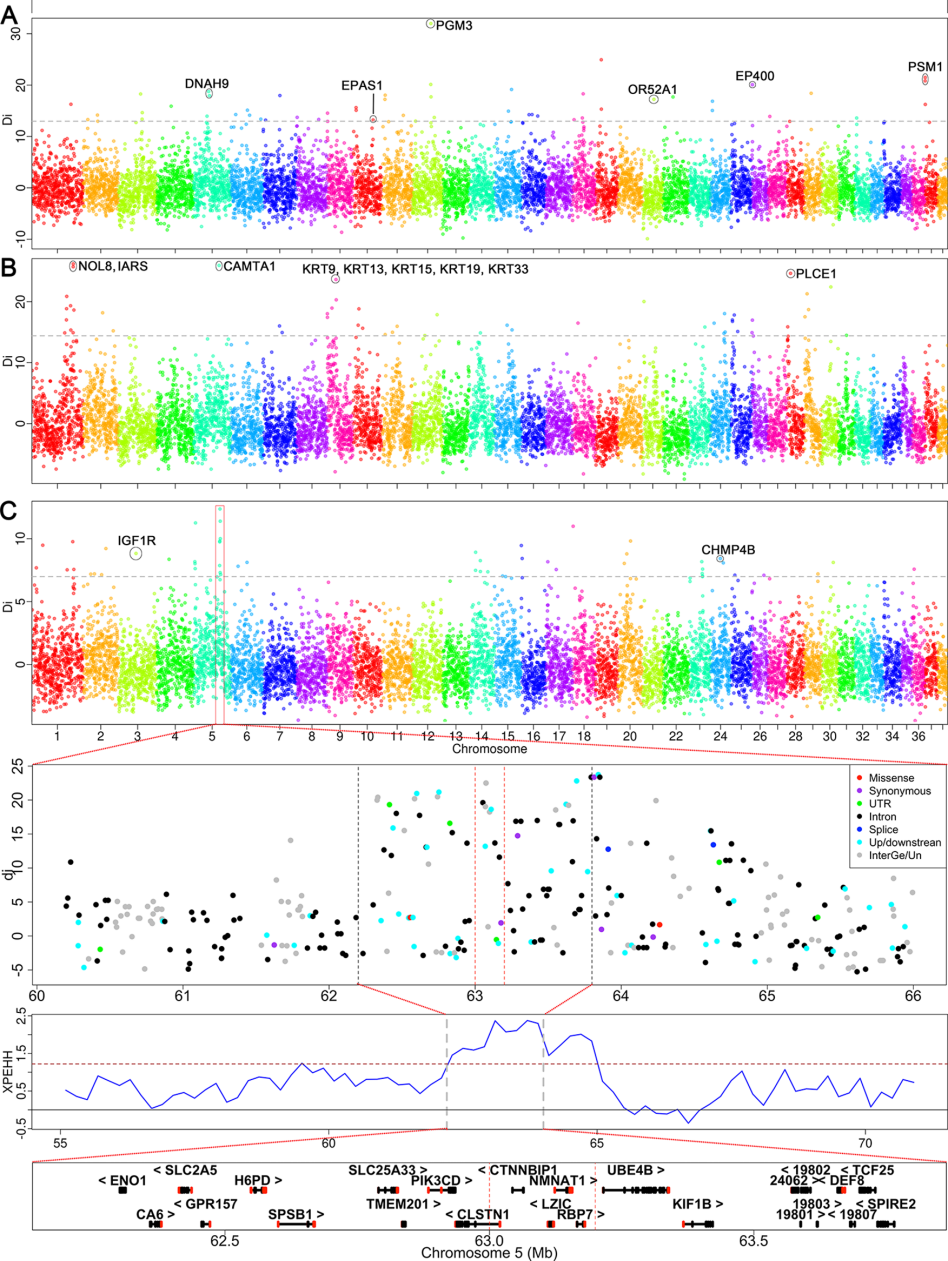
Genome-wide scan for selection signatures in three specific dog groups. The y-axis shows the *d_i_* values of allele frequency difference between the tested group and the control. The analysis was performed using the *d_i_* statistics. The *d_i_* values were calculated for autosomal SNPs in 200-kb windows. The grey dash represents the threshold of 99.5^th^ percent signal level in whole-genome. **(A)** Selection signatures identified in Qinghai-Tibetan Plateau dogs. **(B)** The signals of selection sweep identified in Xi dogs. **(C)** The selection signals detected in mountain hounds. The strongest signal was identified on the CFA 5, where the regional overview of selection signatures and candidate genes are shown below panel 4C. The dashed lines indicate the core region.

We analyzed the signatures of selection in Qinghai-Tibet plateau dogs, Xi dogs, and Mountain hounds to identify selection signals (genes) that may be related to high-altitude adaptation, running speed and hunting ability in mountain areas, respectively (**Supplementary Table S1**). We identified 153 windows containing 1,463 SNPs that we considered the putative signatures of selection in all three groups. The windows showing selection signatures were clustered into 42, 38, and 43 selection regions in the genome of Qinghai-Tibet plateau dogs, Mountain hound dogs, and Xi dogs, respectively (**Figure 4** and **Supplementary Table S5**). We found five selection regions shared between two dog groups, including two regions shared between Qinghai-Tibet plateau and Xi dogs (CFA 11: 8.4–8.6 Mb, 5 SNPs and CFA 26: 0.8–1.0 Mb, 6 SNPs) and three regions between Mountain hounds and Xi dogs (CFA1: 84.4–84.6 Mb, 12 SNPs; CFA 1: 100.4–100.6 Mb, 5 SNPs; and CFA14: 27.0–27.2 Mb, 10 SNPs).

### Candidate Genes That Underwent Selection and Might Be Associated With Phenotypes

We used selection sweep analysis to identify possible candidate genes associated with specific phenotypes of Chinese indigenous dogs.

### Candidate Genes Related to the Adaption of High Altitude

Three breeds (C_HTM, C_TMf, and C_LzD) used in this study are located on the Qinghai-Tibet plateau, which has an altitude >3,000 meters; all non-plateau dogs live in regions with an altitude <1,000 meters above sea level. This enabled us to identify genes that were selected for their ability to adapt to high-altitude in Qinghai-Tibet dogs. In these dogs, we detected 453 SNP outliers. These SNP outliers correspond to 98 candidate genes in 42 genomic regions (24 chromosomes), including *EPAS1*, *DNAH9*, *PGM3*, *EP400*, O*R52A1*, and *PMS1* (**Supplementary Table S5**). The strongest signal was identified at CFA 12: 43.6–43.8 Mb, where *PGM3* is located (**Figure 4A**). We further used the haplotype-based method (cross-population extended haplotype homozygosity, XPEHH) to detect recent or ongoing selection between high and low altitude dogs (**Supplementary Figure S12A**). Consistently, we identified two significant selection signals overlapping with those detected by *di*-statistics, including *EPAS1* and *OR52A1* (**Supplementary Table S5**).

### Candidate Genes Related to Running Speed

Xi dogs are a popular hunting dog, which has been famous for its hunting speed (60 km/h). Chinese Xi dogs have been mainly divided into two sub-populations (Shandong Xi dog and Shaanxi Xi dog), but sometime four sub-populations (Hebei Xi, Mongolia Xi, and the above two). Only the Shandong Xi and Shaanxi Xi dogs were used for selection sweep analysis according to their topological relationship in the NJ- tree described above (**Supplementary Table S1**). We identified a total of 492 SNP outliers showing selection. These SNP outliers are located within 43 genomic regions of 20 chromosomes and correspond to 107 candidate genes (**Supplementary Table S5**). The strongest selection signal was identified at CFA 1: 99.0–99.2 Mb, where *NOL8* and *IARS* genes are located (**Figure 4B**). Interestingly, we detected a remarkable signal both in *di*-statistics and XPEHH analysis, where keratin family genes *KRT9*, *KRT13*, *KRT15*, *KRT19*, and *KRT33* are located (**Figure 4B** and **Supplementary Figure S12B**). Several strong selection signals and interesting candidate genes were also detected at CFA 1: 83.44–83.57 Mb (*RORB*), CFA 5: 60.82–60.91 Mb (*CAMTA1*), and CFA 28: 8.20–8.40 Mb (*PLCE1*) (**Figure 4B**).

### Candidate Genes Related to Hunting Ability in Mountain Areas

Liangshan (C_LSH) and Qingchuan hounds (C_QCH) are mainly distributed in mountainous areas of Western China’s Sichuan province (**Figure 1**). The major characteristics of the two mountain hound breeds are their hunting abilities in mountainous areas (e.g., enhanced ability to see at night, endurance, and aggression). We identified a total of 518 SNP outliers exhibiting selection signals in these mountain hounds. The SNP outliers are located within 38 genomic regions on 19 chromosomes, which contain 106 candidate genes (**Supplementary Table S5**). We found a consecutive selection region on CFA5 involving 7 windows by *di*-statistics (62.2–64.0 Mb) or 13 windows by XPEHH (62.2–65 Mb) (**Figure 4C** and **Supplementary Figure S12C**). The highest *d_i_* statistic window (*d_i_* = 12.36) included four genes *CTNNBIP1*, *LZIC*, *NMNAT1*, and *RBP7* (**Figure 4C**). We also identified selection signals at *IGF1R* (CFA 3: 41.80–41.99 Mb), a gene is associated with body size (Hoopes et al., 2012), and bone growth and density (Yakar et al., 2002).

### Other Important Genes Related to Phenotypes

As described above, the genome-wide scan in each of 13 Chinese dog breeds revealed more than 600 windows that showed signatures of selective sweep (**Supplementary Table S3**). Here, we highlight some genomic regions that showed selection signals and include the genes which have been reported to be the causative genes responsible for dog complex traits (**Table 2**). *RCL1* is associated with canine snout ratio and curly tail (Vaysse et al., 2011). We identified a selection signature at *RCL1* (CFA 1: 93.0–93.2 Mb) in C_SXX. *BMP3* contributes to dog skull diversity (Schoenebeck et al., 2012). We identified the selection signal at the *BMP3* location (CFA 32: 5.2–5.4 Mb) in C_MGX. The gene *MSRB3* affects ear size and type (Vaysse et al., 2011), *RSPO2* affects coat variation (Cadieu et al., 2009), and *KITLG* affects coat color (Ciampolini et al., 2013). We found selection sweep in these three genes (CFA 10: 7.8–8.0 Mb, CFA 13: 8.6–8.8 Mb, and CFA 15: 29.4–29.6 Mb) (**Table 2**).

**Table 2:** The windows showing signatures of selection sweep and including genes responsible for dog phenotypes.

Chr	No. of SNPs	Region (Mb)	*di* value	Gene	Associated trait	Reference	Breed
1	19	93.0-93.4	23.32	*RCL1*	curly tail, snout ratio	(Vaysse et al., 2011)	C_SXX
10	20	7.8-8.2	20.17	*MSRB3*	ear size and type	(Vaysse et al., 2011)	C_QCH, C_SrP, C_TMf, C_XSH, C_CDH, C_CRD, C_GXH, C_LSH
13	13	8.6-8.8	34.44	*RSPO2*	Coat variation	(Cadieu et al., 2009)	C_XSH
15	13	29.4-29.6	20.78	*KITLG*	hematocrit, coat color	(Ciampolini et al., 2013)	C_CDH
32	14	5.2-5.4	21.92	*BMP3*	skull diversity	(Schoenebeck et al., 2012)	C_MGX

Chr, Chromosome; No. of SNPs, the number of SNPs showing selection signature; Region, chromosome region where the SNPs showing selection signature are located. Breed, the dog breeds in which the signatures of selection were detected.

## Discussion

In the current study, we investigated the population structure, genetic diversity, and LD extent of 15 Chinese indigenous dog breeds using the 170K SNP chips. We also identified candidate genes that have undergone selection. To our knowledge, this is a novel study that systematically performs population genetic analysis in multiple Chinese dog breeds.

### Comparison of Genetic Diversity and LD Extent Between Chinese and Western Dog Breeds

From the results of genetic diversity analysis (the parameters of genetic variability), we suggest that Chinese indigenous dogs have higher levels of genetic diversity compared with Western dogs. Our finding could be explained by two primary reasons. First, the domestication of indigenous Chinese dogs occurred much earlier than domestication of Western dogs (Wang et al., 2016). Second, from a historical perspective, we inferred that, just like village dogs, Chinese indigenous dogs might undergo less intensive selection because there was no specific selection purpose during the formation of breeds compared to most modern dog breeds. Consistent with this reasoning, we observed that Chinese dogs and Western dogs had comparable SNP polymorphisms at MAF ≥0.1 (75,684 vs. 70,323).

Most Chinese dogs have shorter-range of LD within populations than Western dogs (**Table 1**). However, Shaanxi Xi dogs (C_SXX) showed the longest-range LD and highest inbreeding coefficient in all Chinese dog breeds, even longer and higher than many Western dog breeds. It is known that the LD extent in a population depends on the history of its effective population size. We inferred that C_SXX might experience population bottleneck in the history that caused a small effective population size. Alternatively, although we collected samples from several unrelated individuals to cover broad consanguinity, our samples could under-represent genomic pool of this breed. Consistent with a previous report (Sutter et al., 2004) and similar to the LD patterns of Western dogs, Chinese dogs have long-range LD within populations and short-range LD across populations. Of note, we observed a much shorter LD extent across populations in Chinese dogs compared to Western dogs (*r^2^*_0.3_ = 8.49 kb vs. 15.07 kb). As described above, domesticating and breeding of Chinese indigenous dogs were much earlier than in Western dogs. We propose that many more generations of recombination have resulted in shorter identical-by-descent chromosome segments across populations.

### Genetic and Population Structure Among Chinese and Western Dogs

In both PCA and NJ analyses, most Western dogs clustered together in one group and Chinese dogs cluster together in separate group. Furthermore, Chinese and Western dogs represent different lineages in ADMIXTURE analyses. However, Greenland sledge dogs (W_GSl) were clearly clustered with Asian grey wolf, Chinese Southwestern dogs (e.g., C_LSH; NJ tree), and Northwestern plateau dogs (C_TMf and C_HTM; PCA). Papillon (W_Pap) and the European Eurasier (W_Eur) dogs were grouped with Chinese Pekingese (C_Pkg). Chinese Kazakhstan shepherd (C_KzS) and Mongolia Xi (C_MGX) dogs from Northwest China were separated from the main Chinese dog clade and were close to Finnish Spitz (W_FSp) and Elkhound (W_Elk) groups (**Figures 2** and **3** and **Supplementary Figure S2**). These results suggested a historical introgression of Western dog breeds into Chinese Kazakhstan shepherd (C_KzS) and Mongolia Xi (C_MGX) dogs, perhaps a result of the immigration of Chinese dogs along the old “silk road” (**Supplementary Figure S7** and **Table S2**). Some Western dogs, such as the Greenland sledge (W_GSl), Papillon (W_Pap), and European Eurasier (W_Eur), indicate descent from Chinese dogs, perhaps a result of immigrating to Europe along the trails from Siberia to Northern Europe (**Supplementary Figures S4**–**S7** and **Table S2**). Another explanation for this observation is that these dogs are recent crossbreeds with indigenous Chinese dogs (e.g., the European Eurasier is a recent crossbreed between the German Wolfspitzes and Chinese Chow Chows). This hypothesis was supported by the ADMIXTURE, TreeMix, and ABBA-BABA analysis, which obtained similar results as the NJ-tree analysis.

### Candidate Genes Possibly Associated With Specific Phenotypes in Chinese Dogs by Selection Sweep Analysis

Natural and artificial selection has dramatically shaped dog genomic variability during domesticating and forming breeds. In this study, we detected selection signatures in the dog genome using statistical *d_i_* values in each of individual breeds and three additional breed groups. The analysis allowed us to identify possible candidate genes for specific phenotypes.

Tibetan mastiff (including C_HTM and C_TMf) and Linzhi Dogs (C_LzD) have lived in the Qinghai-Tibetan plateau for centuries, which has resulted in high-altitude adaptations. This fact helps us identify candidate genes for high-altitude adaptation through selection sweep analysis. Our study supports the finding that *EPAS1* is the responsible gene for adapting to the high-altitude (Yi et al., 2010; Gou et al., 2014). We also identified *DNAH9* which is located within another region showing selection signatures, as a candidate gene associated with high-altitude adaptation, which has also been reported in Ethiopian sheep (Edea et al., 2019). Several genes associated with hematopoiesis (*PGM3*) (Greig et al., 2007), erythropoiesis (*EP400*) (Fujii et al., 2010), and heart morphology (*PMS1*) (Bult et al., 2019) are located within the regions where we found strong selection signals. These genes are important candidate genes for high-altitude adaptation of dogs (**Figure 4A** and **Supplementary Table S5**).

Running speed, endurance, and aggression are important characteristics for hunting dogs and hounds. They are the result of strong artificial selection. Chinese Xi dogs are famous for fast running and endurance in hunting. We identified several candidate genes that may be responsible for dog hunting ability. *NOL8* is located within the region showing the strongest selection signals. This gene is associated with fasting circulating glucose levels (Bult et al., 2019). Circulating glucose provides energy for endurance. *KRT9* affects the structure of the suprabasal layers of the palmoplantar epidermis and footpad morphology (Fu et al., 2014). *RORB* is involved in the processing of sensory information, and *RORB* knock-out mice show impaired limb coordination, degenerated retinas, and leads to *vacillans* phenotype (Andre et al., 1998). The second strongest selection signal we identified in Xi dogs was *CAMTA1*. *CAMTA1*. Knock-out mice exhibit impaired coordination (Long et al., 2014). Another candidate gene, *PLCE1*, is associated with the heart’s stress response (Zhang et al., 2013).

C_LSH and C_QCH are the predominant hounds for people living in the mountainous region of Sichuan Province. Strong artificial selection for hunting abilities of these two dog breeds is expected to present selection signatures in the genome. We identified several candidate genes possibly responsible for night vision and endurance. *NMNAT1* is involved in retinal vasculature morphology (Greenwald et al., 2016). *RBP7* encodes protein binding all-trans-retinol and is required for vitamin A stability and metabolism (Folli et al., 2002). *SLC2A5* influences lens and retina morphology in mice (MGI) (Bult et al., 2019). These three genes may affect night vision capability of mountain hounds. *H6PD* is related to skeletal muscle fiber morphology and glycogen levels (Semjonous et al., 2011), which may influence the endurance of mountain hounds. However, the possible relationships of all genes showing signals of selection described above with dog phenotypes were inferred from gene functions reported previously in dogs or other animals. Further experiments will be needed performing to confirm the causality of these genes with the phenotypes in the future studies.

In summary, we systematically investigated genetic diversity, genomic and population structure, and differentiation of multiple Chinese indigenous dog breeds. We present a comprehensive comparison between Chinese and Western dog breeds using the CanineHD 170K SNP BeadChip. We also identified interesting genes that might be responsible for specific phenotypic variations of Chinese indigenous dogs by analyzing genome-wide selection signatures. Furthermore, this study provides important knowledge about the current population and genomic structure of Chinese indigenous dog breeds. These results reinforce the conclusion that Chinese indigenous dogs with great variations of phenotypes are important resources for identifying genes responsible for complex traits. What's more, the recent availability of multiple whole-genome datasets from different dog breeds including several Chinese dog breeds (Parker et al., 2017) will allow us to further compare genomic structure of Chinese indigenous dog breeds with other dog breeds in the future study.

## Data Availability Statement

The datasets generated for this study can be found in the Dryad database; https://datadryad.org/stash/share/u4FKRNZ4wueHyQEnYTeZ59XAWvuVg-aFMhTwqr1gfB4.

## Ethics Statement

All works referring to experimental animals were conducted according to the guidelines for the care and use of experimental animals established by the Ministry of Agriculture of China. Animal Care and Use Committee (ACUC) in Jiangxi Agricultural University approved this study.

## Author Contributions

LH conceived and designed the experiments and revised the manuscript. CC designed the experiments, analyzed the data, wrote, and revised the manuscript. QY performed the experiments, analyzed the data, and wrote the manuscript. HC analyzed the data and wrote the manuscript. JY, CL, and RW performed the experiments.

## Funding

This work was supported by The National Key Research and Development Program of China (2016YFD0501008) and Jiangxi Provincial Program for cultivating young scientist (2010BQ01500).

## Conflict of Interest

The authors declare that the research was conducted in the absence of any commercial or financial relationships that could be construed as a potential conflict of interest.

## Supplementary Material

Figure S1 Genetic distance estimated within each of Chinese and Western dog populations. The distance estimated by PLINK (v1.9) with the formula: , Where IBS1 and IBS2 are the number of loci that share one or two alleles identical by state (IBS), respectively, and N is the number of loci tested. Only abbreviated names are shown in the figure. The full name of each breed is listed in **Table 1**.

Figure S2 The neighbor-joining tree of Chinese and Western dog breeds based on genome-wide allele sharing. The black clades represent Western dogs, and the colorful clades represent Chinese dogs.

Figure S3 Decline in genome-wide linkage disequilibrium (LD) estimated in the Western dog breeds.

Figure S4-S7 Genetic relationships and admixture amongst Chinese and Western dog populations inferred using the TreeMix program with 0-7 migration edges. C_AGW was used as an outgroup to root the tree. Migration arrows are colored according to their weight. Horizontal branch lengths are proportional to the amount of genetic drift that has occurred on each branch. The scale bar shows ten times of average standard error (s.e.) of the entries in the sample covariance matrix. The colored dash in the phylogenetic tree represents different genetic groups.

Figure S8 The population differentiation between pair-wise groups. Pair-wise Fst values were estimated for all autosomal informative SNPs.

Figure S9-S11 Genome-wide scan for selection signatures in each of 13 Chinese dog breeds. The y-axis shows the di values of allele frequency difference and iHS values of extended homozygosity haplotype. The analysis was performed for autosomal SNPs in 200-kb windows using the di statistics and Selscan software. The interesting candidate genes are also indicated in the figures.

Figure S12 Genome-wide scan for selection signatures in three dog populations of Qinghai-Tibet plateau dogs, Xi dogs and Mountain hounds to identify selection signals that may be related to high-altitude adaptation, running speed and hunting ability in mountain, respectively. The y-axis shows xpehh values of extended homozygosity haplotype. The analysis was performed for autosomal SNPs in 200-kb windows using Selscan software. The interesting candidate genes are also indicated in the figures.

Table S1 Three specific dog groups used for detecting signatures of selection.

Table S2 Details about gene flow between Chinese and Western dog populations from ABBA-BABA analyses.

Table S3 The windows showing selection signatures identified in each of Chinese indigenous dog breeds.

Table S4 GO functional enrichment and KEGG pathway analysis for genes located within Selection regions in each of Chinese dog populations.

Table S5 Annotation of SNPs showing signatures of selection in three specific dog groups.
