# Supplementary figures and images for: Genetic Diversity and Signatures of Selection in 15 Chinese Indigenous Dog Breeds Revealed by Genome-Wide SNPs
Source: Front Genet. 2019 Nov 15;10:1174. doi: 10.3389/fgene.2019.01174 (PMC6872681; doi:10.3389/fgene.2019.01174)

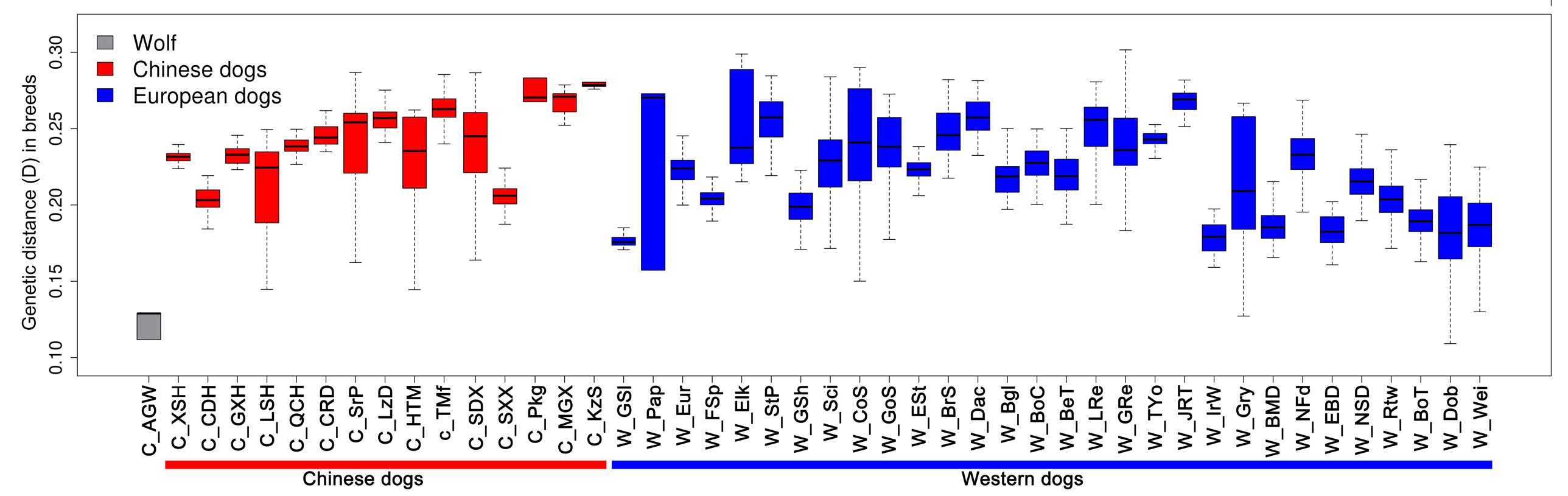

Supplement: Figure S1 — Genetic distance estimated within each of Chinese and Western dog populations. The distance estimated by PLINK (v1.9) with the formula: , Where IBS1 and IBS2 are the number of loci that share one or two alleles identical by state (IBS), respectively, and N is the number of loci tested. Only abbreviated names are shown in the figure. The full name of each breed is listed in Table 1. [file Image_1.tif]

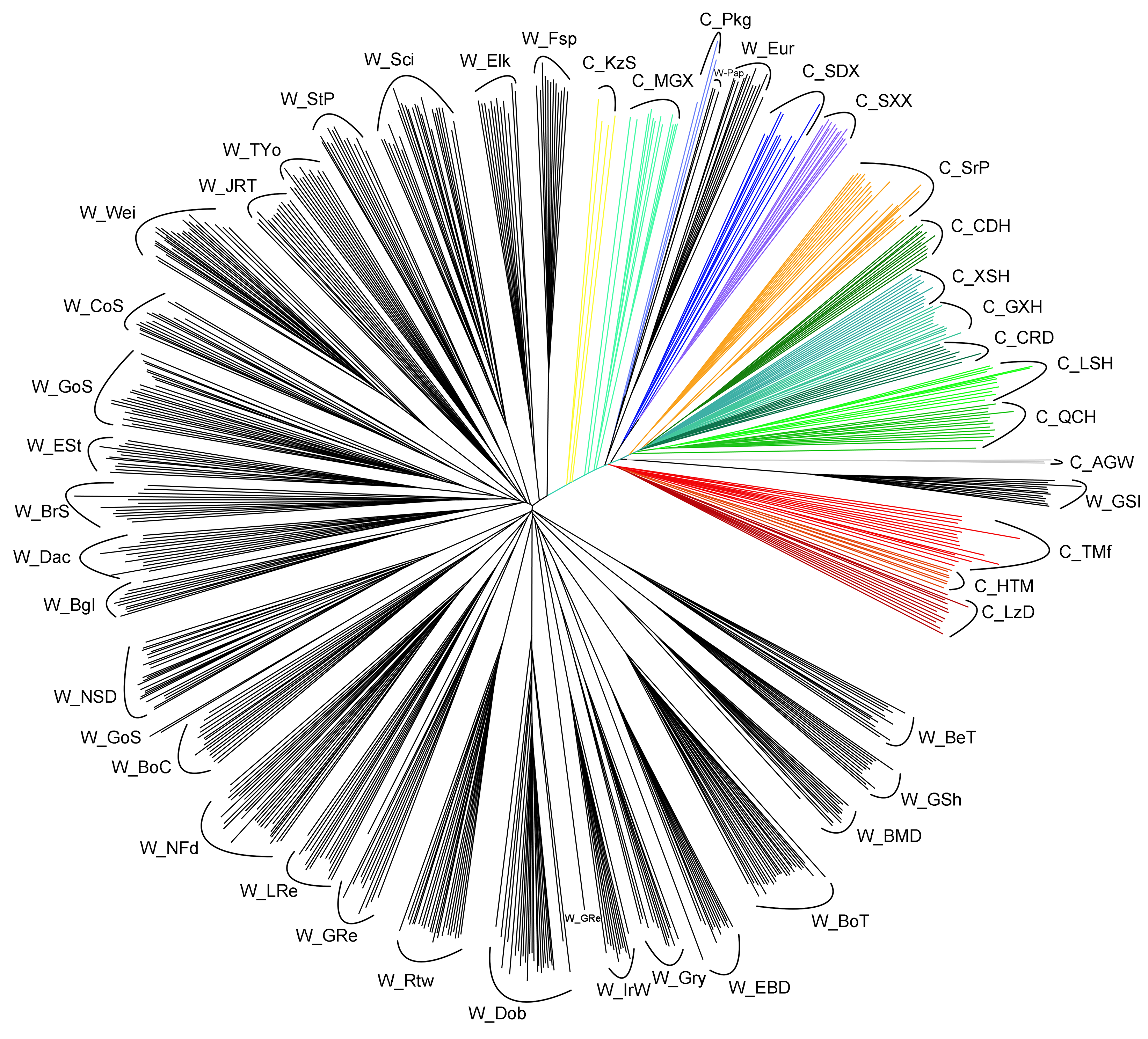

Supplement: Figure S2 — The neighbor-joining tree of Chinese and Western dog breeds based on genome-wide allele sharing. The black clades represent Western dogs, and the colorful clades represent Chinese dogs. [file Image_2.tif]

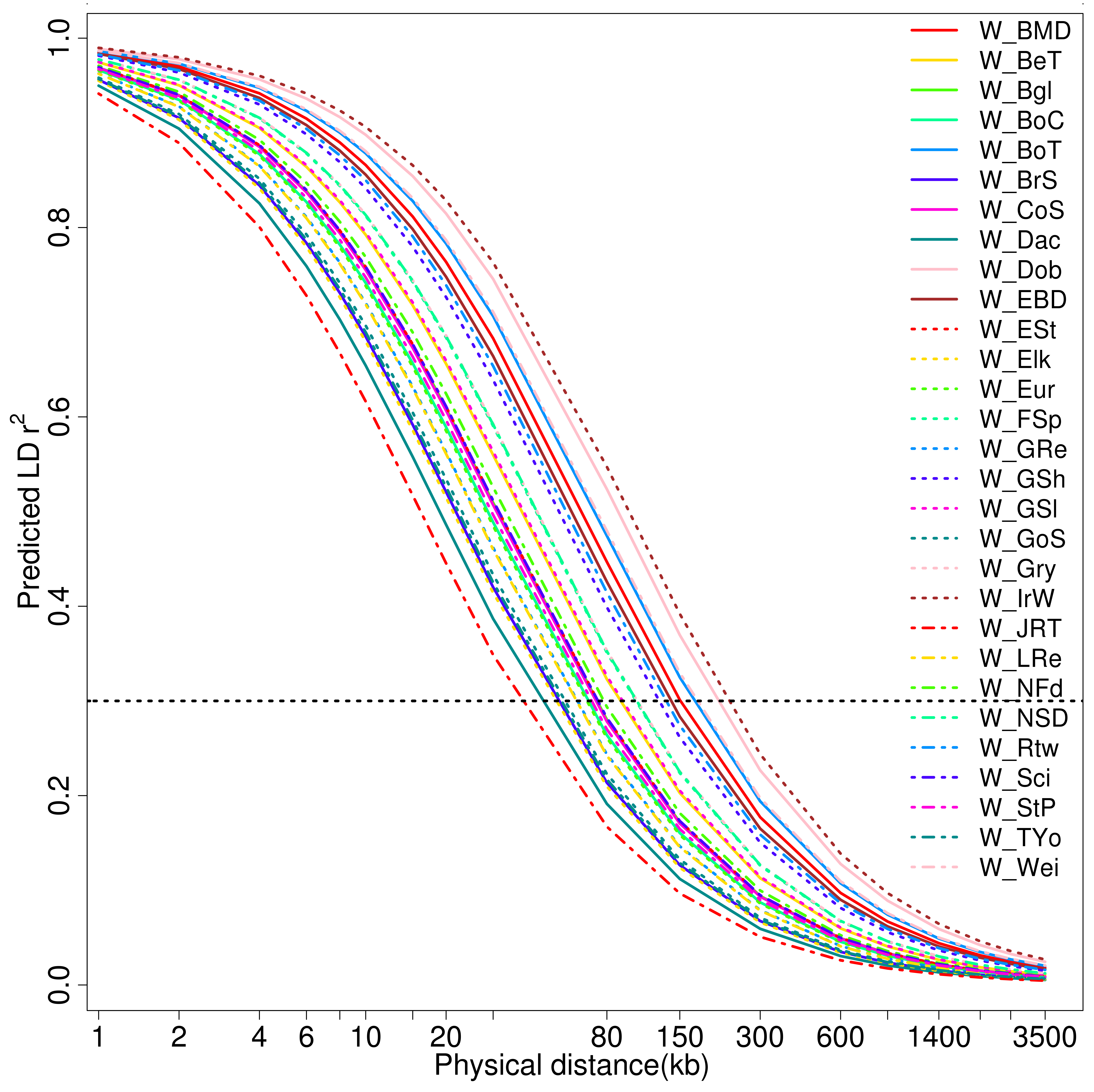

Supplement: Figure S3 — Decline in genome-wide linkage disequilibrium (LD) estimated in the Western dog breeds. [file Image_3.tif]

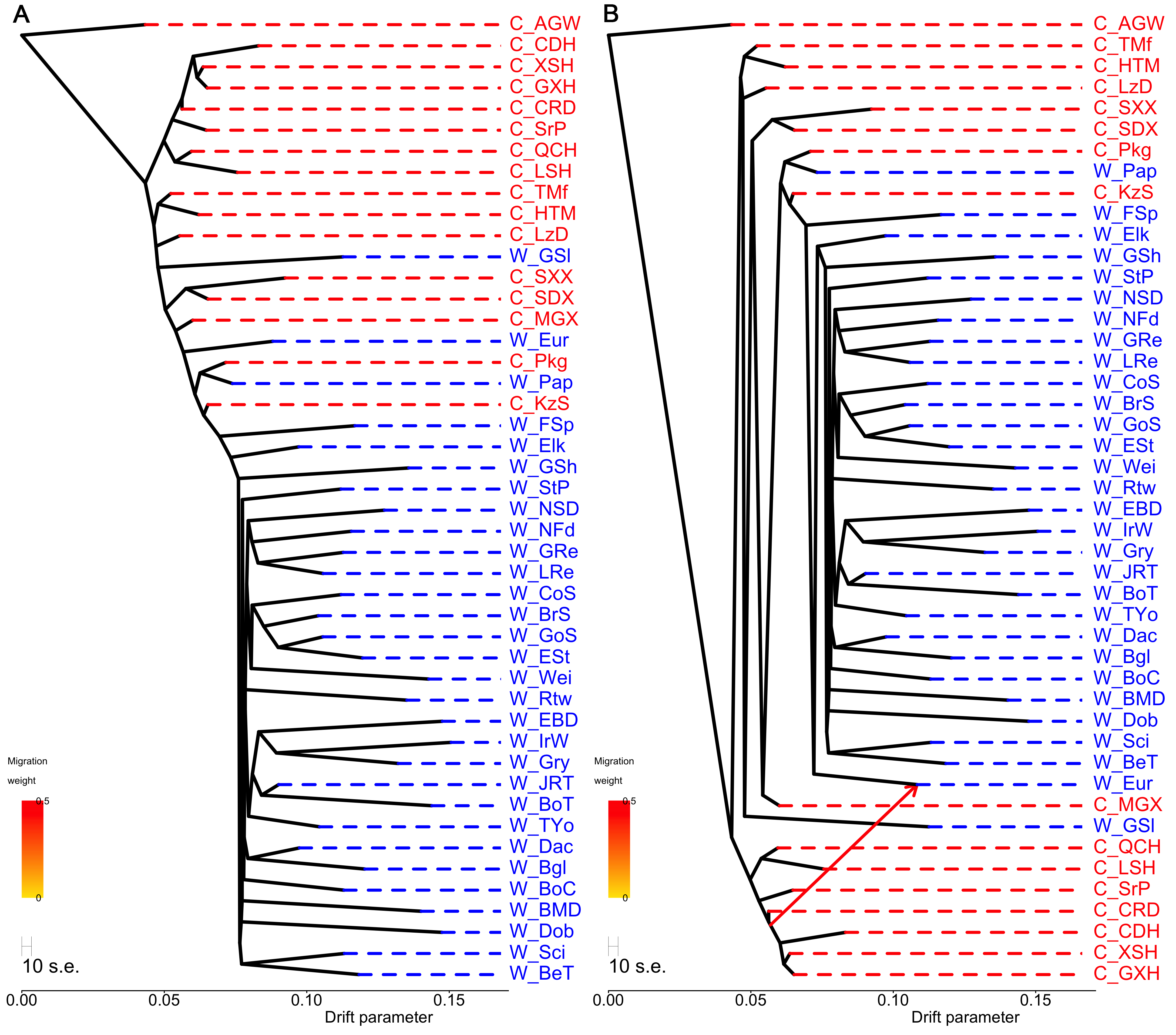

Supplement: Figure S4-S7 — Genetic relationships and admixture amongst Chinese and Western dog populations inferred using the TreeMix program with 0-7 migration edges. C_AGW was used as an outgroup to root the tree. Migration arrows are colored according to their weight. Horizontal branch lengths are proportional to the amount of genetic drift that has occurred on each branch. The scale bar shows ten times of average standard error (s.e.) of the entries in the sample covariance matrix. The colored dash in the phylogenetic tree represents different genetic groups. [file Image_4.tif]

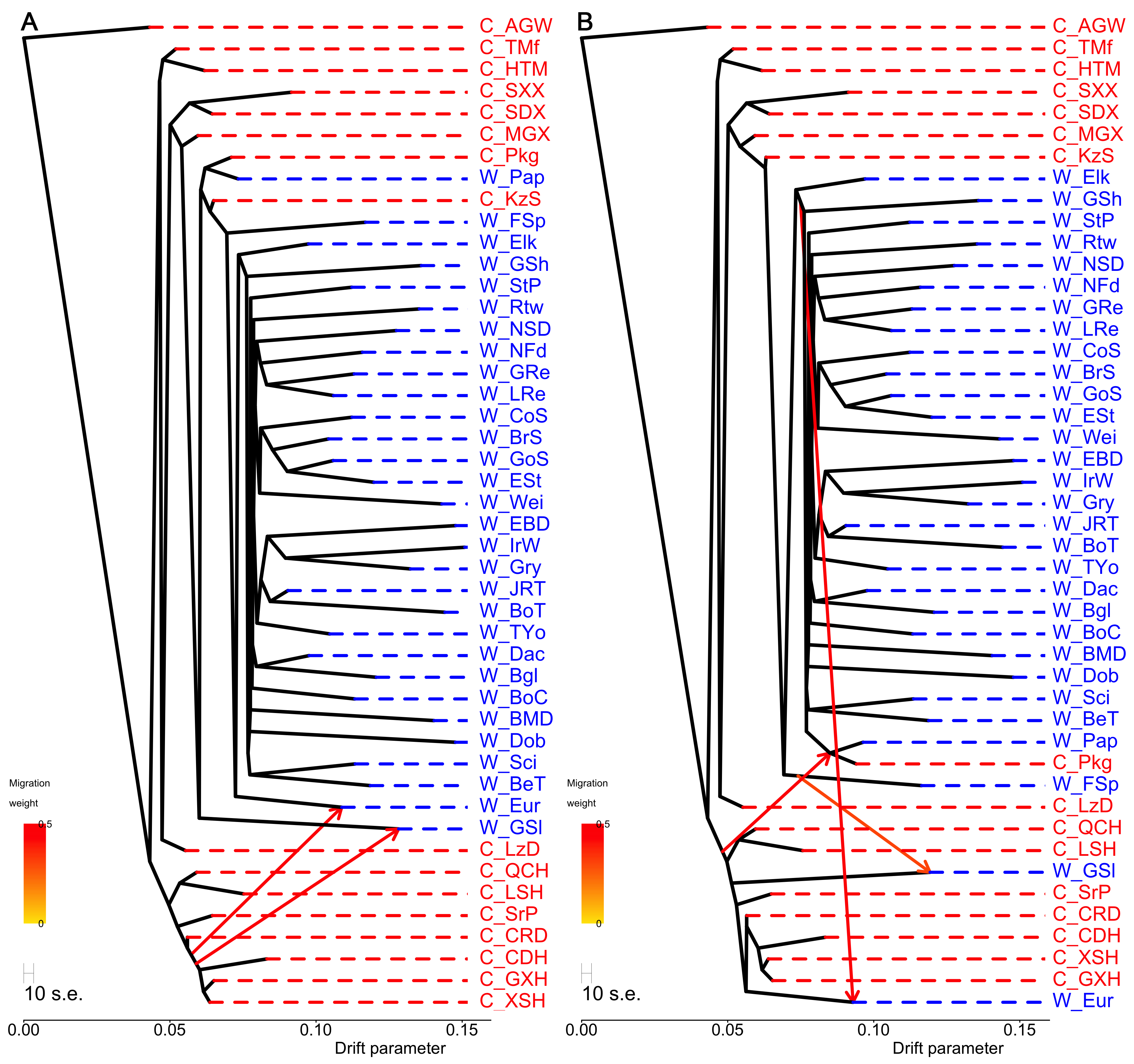

Supplement: Supplementary file 5 [file Image_5.tif]

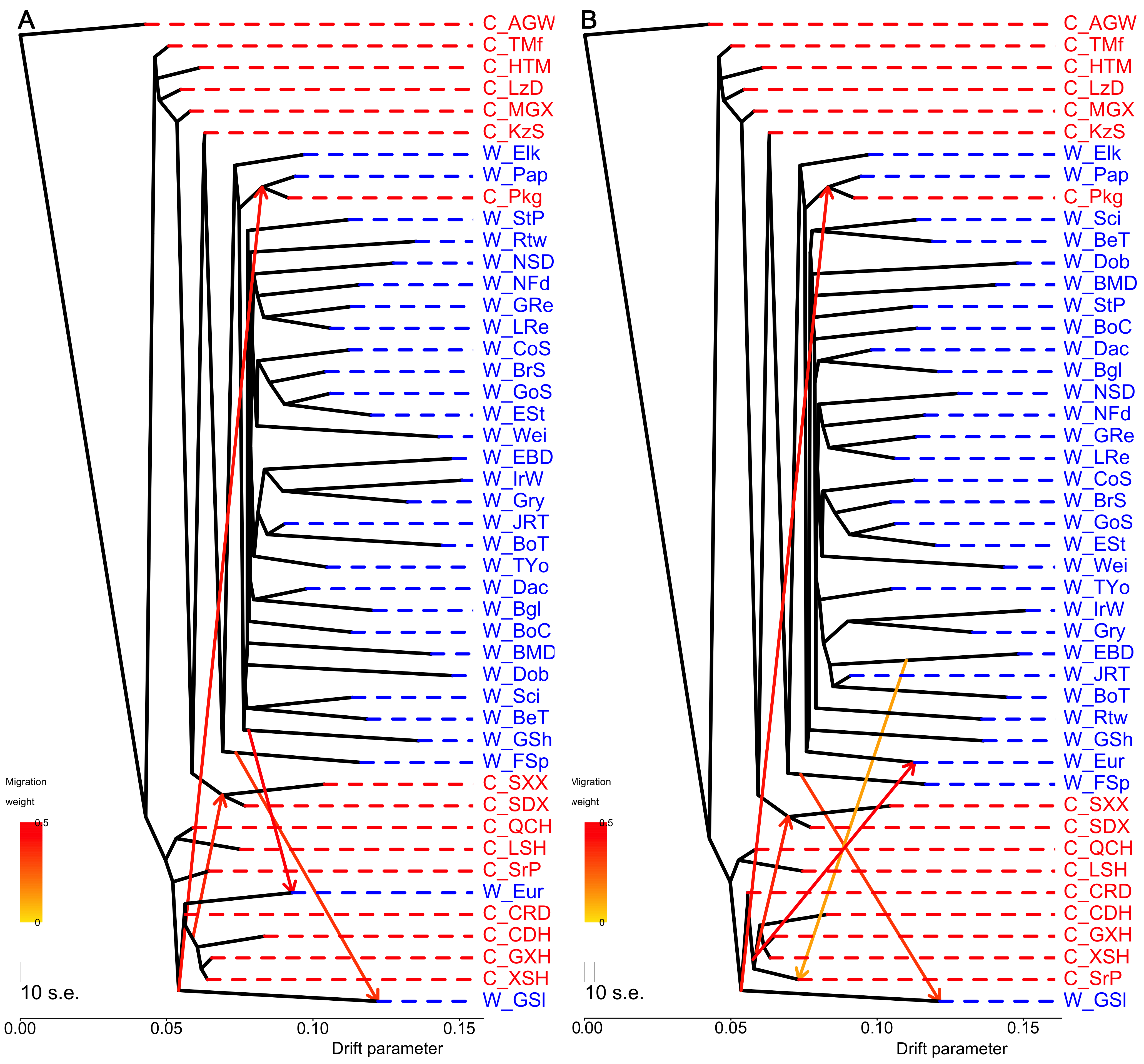

Supplement: Supplementary file 6 [file Image_6.tif]

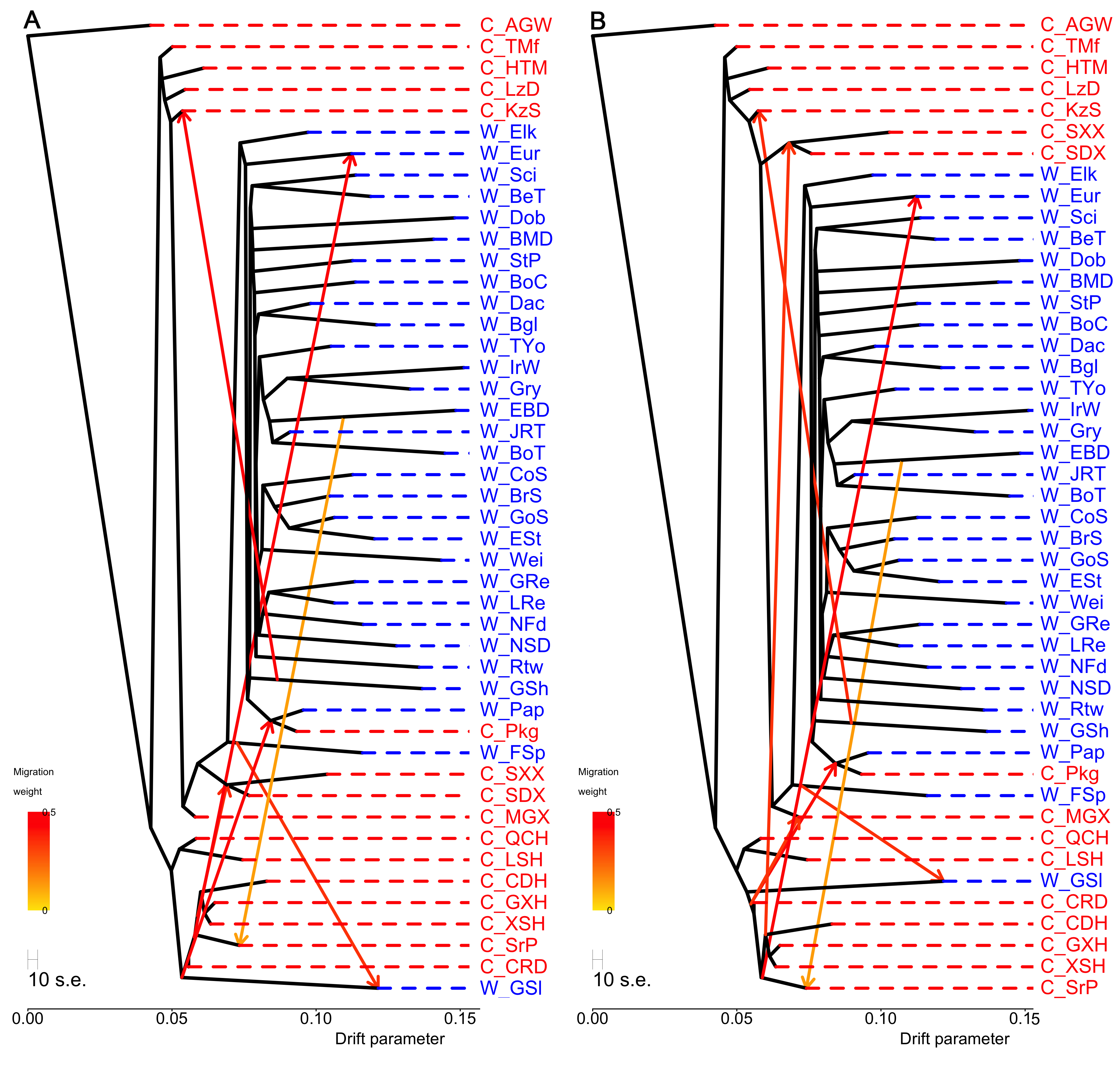

Supplement: Supplementary file 7 [file Image_7.tif]

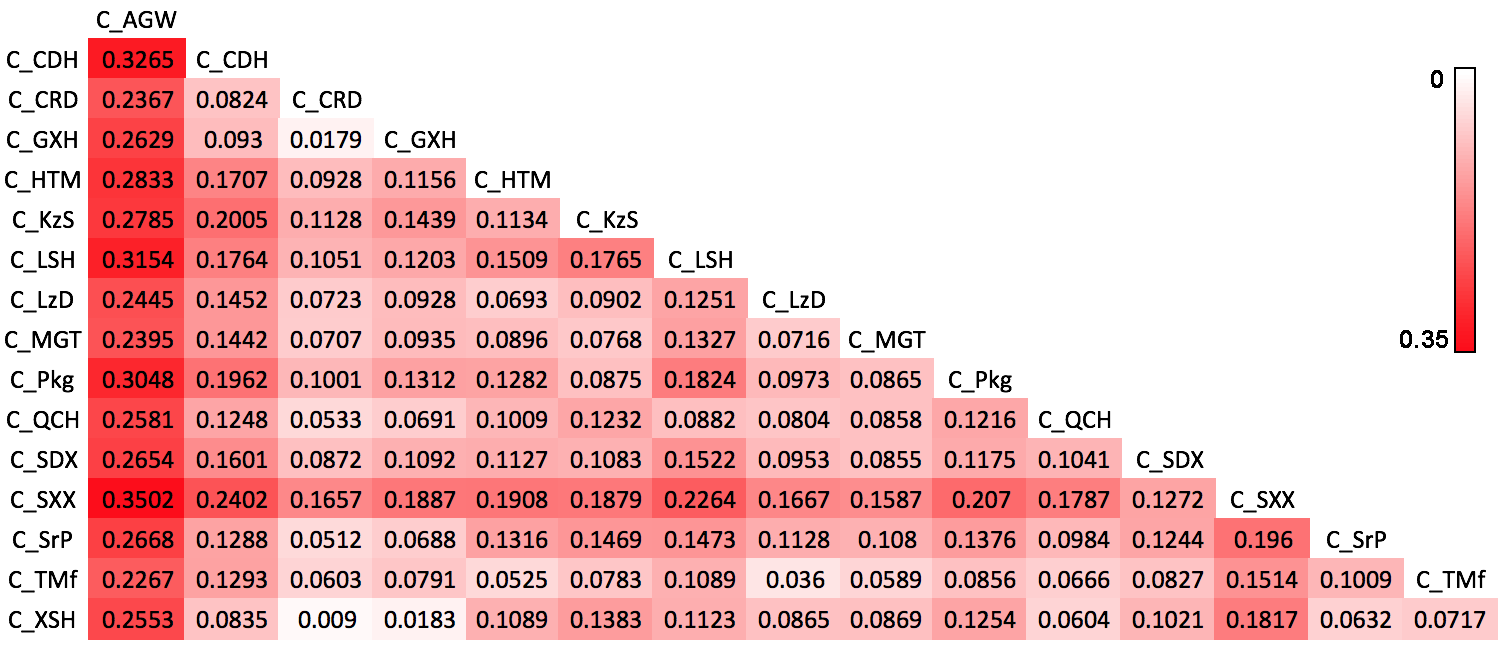

Supplement: Figure S8 — The population differentiation between pair-wise groups. Pair-wise Fst values were estimated for all autosomal informative SNPs. [file Image_8.tif]

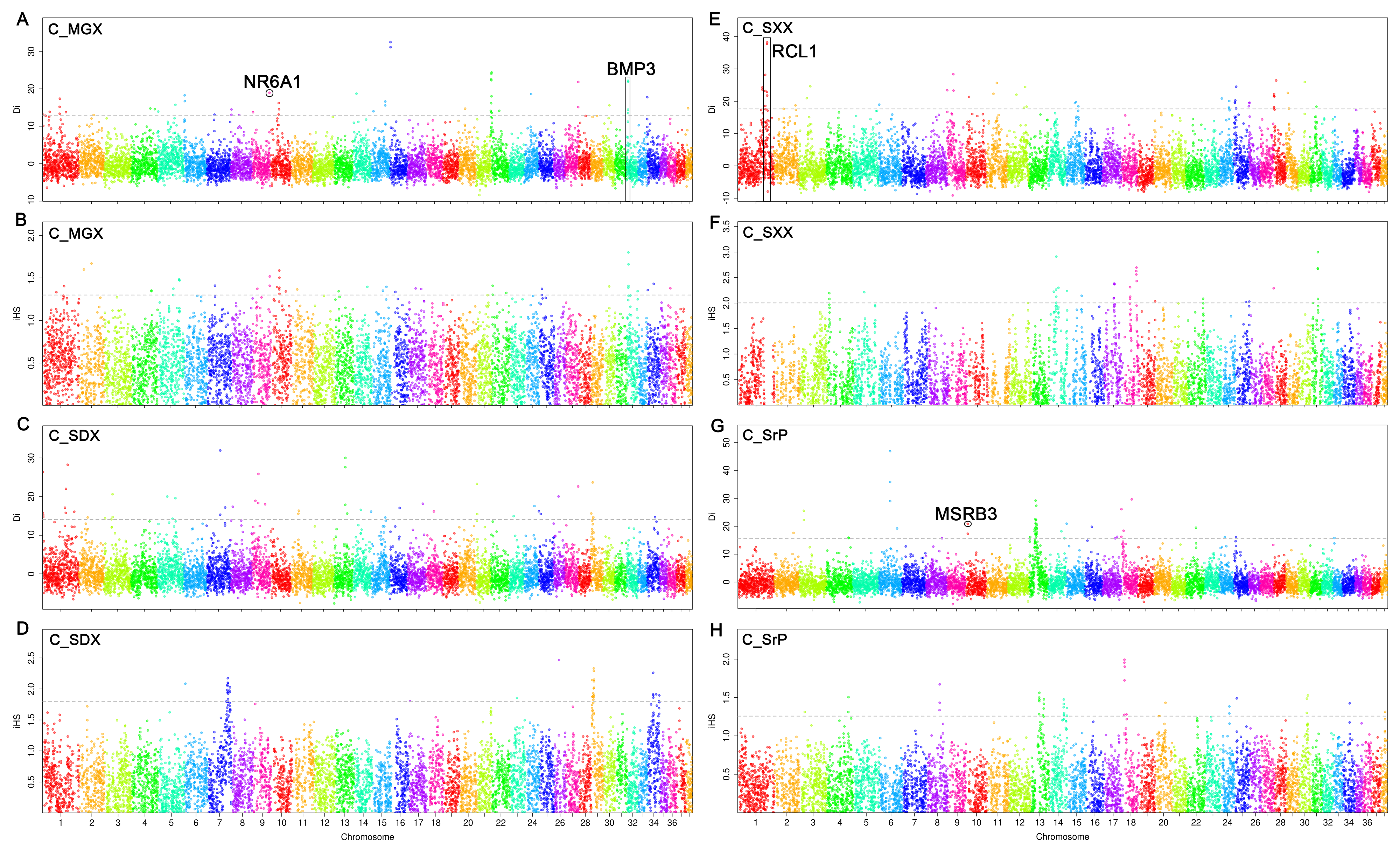

Supplement: Figure S9-S11 — Genome-wide scan for selection signatures in each of 13 Chinese dog breeds. The y-axis shows the di values of allele frequency difference and iHS values of extended homozygosity haplotype. The analysis was performed for autosomal SNPs in 200-kb windows using the di statistics and Selscan software. The interesting candidate genes are also indicated in the figures. [file Image_9.png]

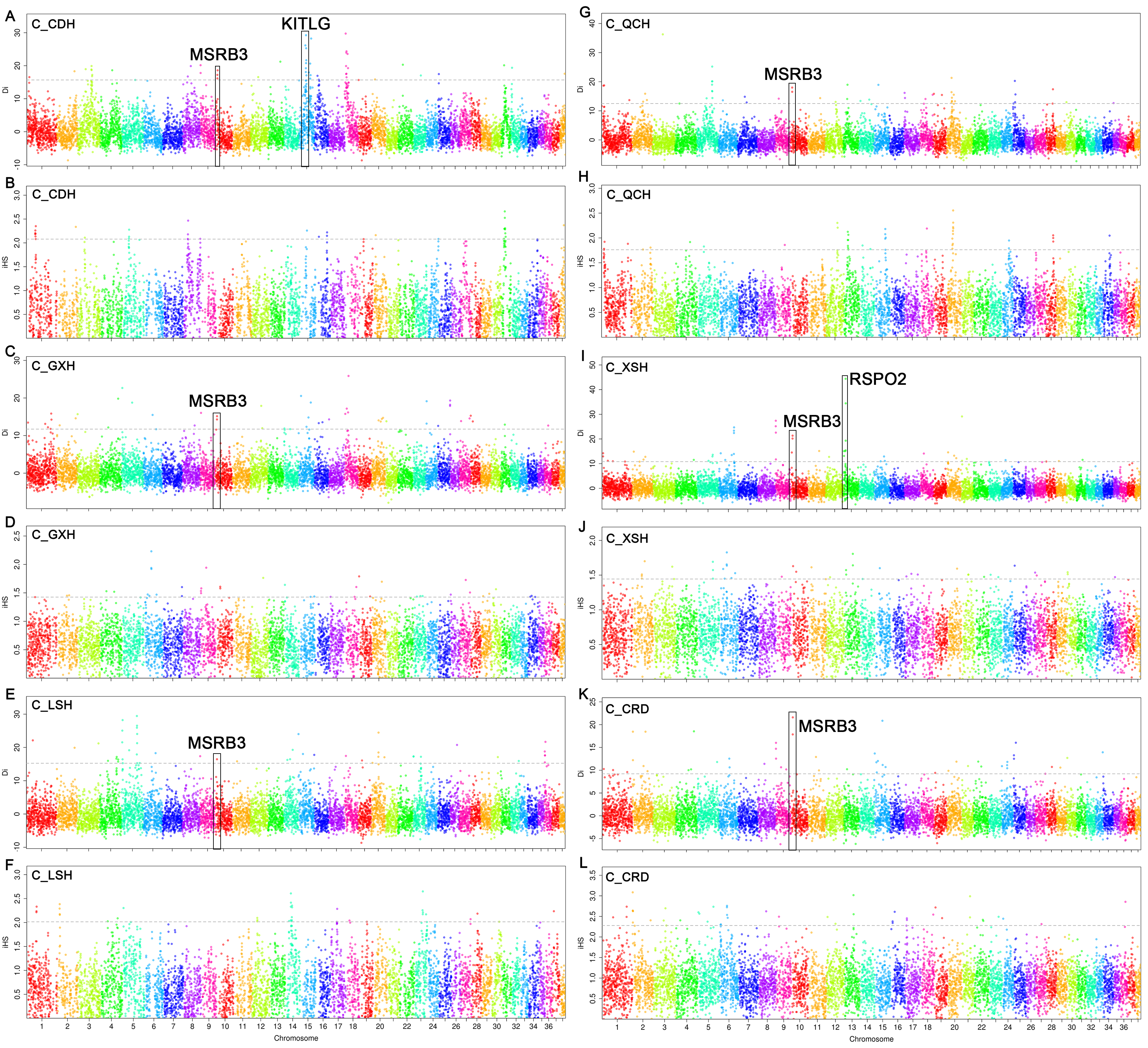

Supplement: Supplementary file 10 [file Image_10.tif]

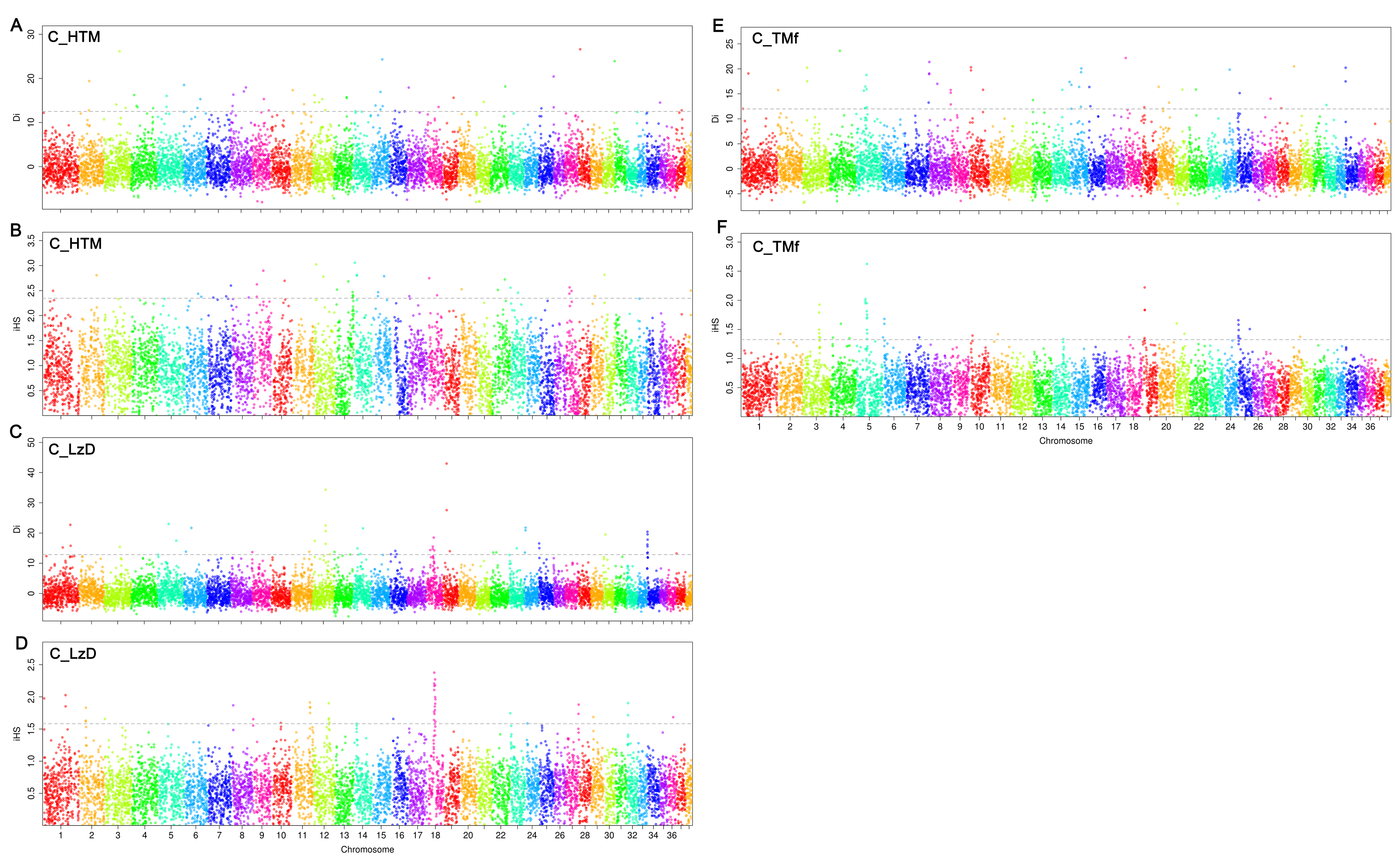

Supplement: Supplementary file 11 [file Image_11.tif]

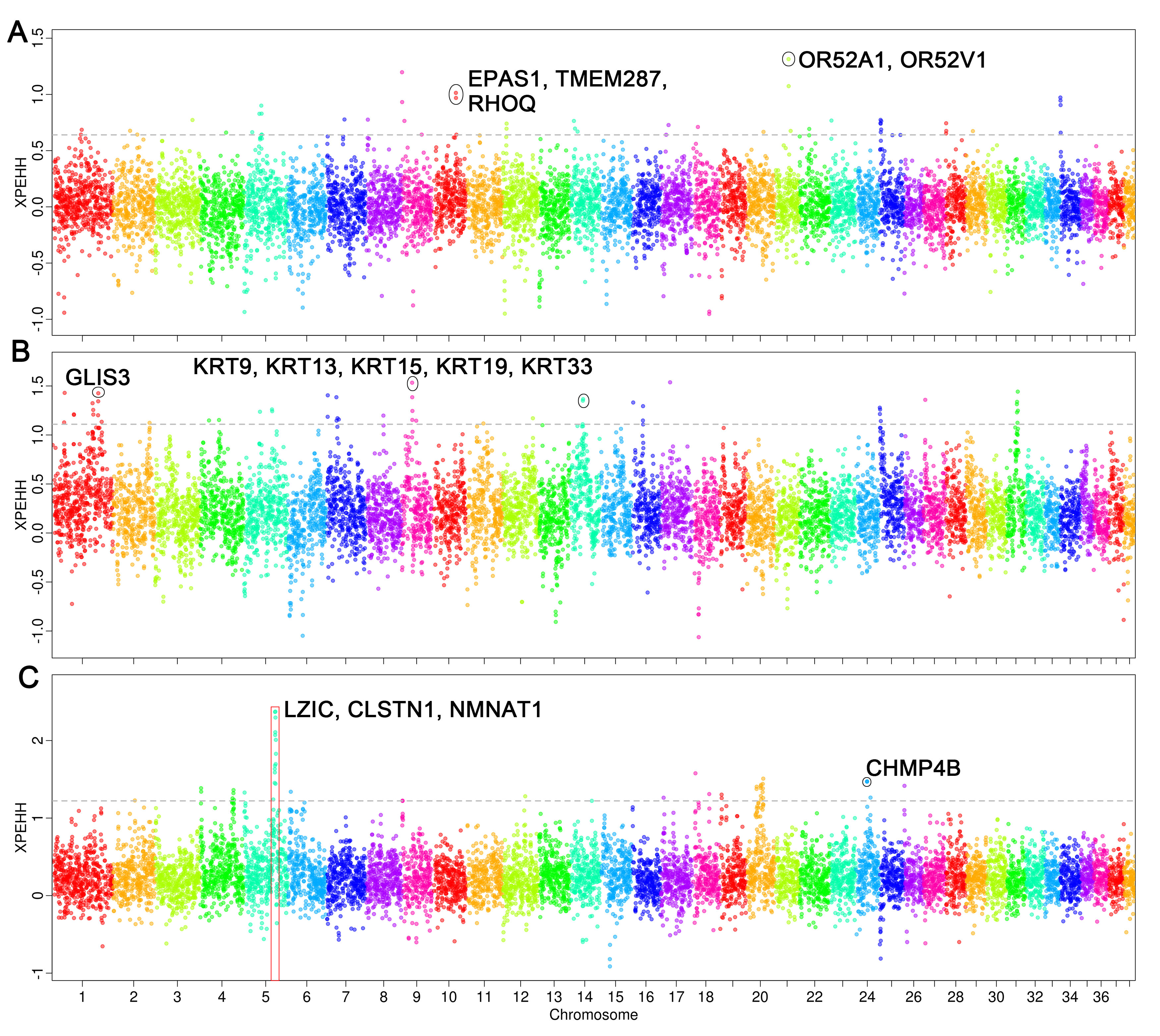

Supplement: Figure S12 — Genome-wide scan for selection signatures in three dog populations of Qinghai-Tibet plateau dogs, Xi dogs and Mountain hounds to identify selection signals that may be related to high-altitude adaptation, running speed and hunting ability in mountain, respectively. The y-axis shows xpehh values of extended homozygosity haplotype. The analysis was performed for autosomal SNPs in 200-kb windows using Selscan software. The interesting candidate genes are also indicated in the figures. [file Image_12.tif]
