# Supplementary material for: Genetic Diversity and Signatures of Selection in 15 Chinese Indigenous Dog Breeds Revealed by Genome-Wide SNPs
Source: Front Genet. 2019 Nov 15;10:1174. doi: 10.3389/fgene.2019.01174 (PMC6872681; doi:10.3389/fgene.2019.01174)
Supplement: Table S1 — Three specific dog groups used for detecting signatures of selection. [file Table_1.doc]

**Table S1**. Three specific dog groups used for detecting signatures of selection

| **Group** | **Main Characteristics** | **Breeds in tested group** | **Breeds in control group** |
| --- | --- | --- | --- |
| Qinghai-Tibetan Plateau dogs | High altitude adaption | Tibetan mastiff (C_HTM, C_TMf),  Linzhi dog (C_LzD) | C_CDH, C_KzS, C_MGX, C_CRD, C_GXH, C_LSH, C_Pkg, C_QCH, C_SDX, C_SrP, C_SXX, C_XSH |
| Xi dogs | Excellent hunting dogs  with fast running speed | Shandong Xi (C_SDX)  Shaanxi Xi (C_SXX) | C_CDH, C_KzS, C_CRD, C_GXH, C_TMf, C_LSH, C_Pkg, C_QCH, C_SrP, C_LzD, C_XSH |
| Mountain hound | Mountain hounds,  good hunting ability | Liangshan Hound (C_LSH)  Qingchuan Hound (C_QCH) | C_KzS, C_MGX, C_TMf, C_Pkg, C_LzD, C_SDX, C_SXX |
